# MITF regulates autophagy and extracellular vesicle cargo in gastrointestinal stromal tumors

**DOI:** 10.1186/s43556-025-00329-9

**Published:** 2025-10-31

**Authors:** Elizabeth Proaño-Pérez, Eva Serrano-Candelas, Mario Guerrero, David Gómez-Peregrina, Carlos Llorens, Beatriz Soriano, Ana Gámez-Valero, Marina Herrero-Lorenzo, Eulalia Martí, César Serrano, Margarita Martin

**Affiliations:** 1 Biochemistry and Molecular Biology Unit, Biomedicine Department, Faculty of Medicine and Health Sciences, University of Barcelona, Barcelona, 08036 Spain; 2 Multidisciplinary and translational research in inflammation and immunoallergy (METRI2 A), Institut d’Investigacions Biomediques August Pi I Sunyer (IDIBAPS), Barcelona, 08036 Spain; 3 Facultad de Ciencias de La Salud, Universidad Técnica de Ambato, Ambato, 180105 Ecuador; 4 Nutrigenx. Facultad de Ciencias de La Salud, Universidad Técnica de Ambato, Ambato, 180105 Ecuador; 5 ProtoQSAR SL, Centro Europeo de Empresas Innovadoras (CEEI), Parque Tecnológico de Valencia, Paterna, Valencia, 46980 Spain; 6 Sarcoma Translational Research Program, Vall d’Hebron Institute of Oncology (VHIO), Hospital Universitario Vall d’Hebron, Vall d’Hebron Barcelona Hospital Campus, C/Natzaret, 115-117, Barcelona, 08035 Spain; 7 Biotechvana, Parque Científico Universidad de Valencia, Paterna, Valencia, 46980 Spain; 8 Department of Medical Oncology, Vall d’Hebron University Hospital, Barcelona, 08035 Spain; 9 Moldrug AI Systems SL, Parque Tecnológico de Valencia, Valencia, 46980 Spain

**Keywords:** MITF, ChIP-seq, Autophagy, Extracellular vesicles, Gastrointestinal stromal tumors (GIST)

## Abstract

The role of Microphthalmia-associated Transcription Factor (MITF) in gastrointestinal stromal tumors (GISTs) remains unclear, although previous studies suggest it contributes to tumor growth regulation. Previously, we demonstrated that MITF depletion reduces GIST cell proliferation and viability, accompanied by decreased expression of BCL-2 and CDK2. To elucidate the mechanisms underlying MITF function in GISTs, we performed chromatin immunoprecipitation and sequencing (ChIP-seq) as well as RNA sequencing. Integrated analyses revealed that MITF directly regulates genes involved in lysosome biogenesis, vesicle trafficking, autophagy, and the mTOR signaling pathway. Transcriptomic profiling following MITF silencing further demonstrated enrichment of differentially expressed genes in PI3K/ mTOR signaling, with downstream effects on tumor growth and autophagy. We next examined the functional consequences of MITF loss on mTOR inhibition-induced autophagy and on extracellular vesicle (EV) content and secretion, given their known interplay in tumor progression. MITF depletion reduced LC3-II levels and impaired autophagy flux, confirming its role in regulating autophagy in GISTs. EV size and number remained unaffected; however, silencing MITF altered EV cargo and notably decreased KIT expression in both cells and EVs. As KIT-containing EVs have been implicated in GIST invasion, these findings suggest that MITF contributes to tumor progression through coordinated regulation of autophagy and EV-mediated signaling. Collectively, our results identify MITF as a key regulator of GIST biology, highlighting its potential as a therapeutic target to limit tumor growth and metastasis.

**Supplementary Information:**

The online version contains supplementary material available at 10.1186/s43556-025-00329-9.

## Introduction

Microphthalmia-associated transcription factor (MITF) is a member of the MiT/TFE family of transcription factors, which also includes TFEB, TFE3, and TFEC [1]. These evolutionarily conserved proteins can form homo- and heterodimers and bind, through their basic domains, to E-box (CANNTG) motifs in target gene regulatory regions [2, 3]. MiT/TFE factors are regulated at both transcriptional and posttranscriptional levels [4–6] and participate in diverse physiological processes such as cell survival, differentiation, metabolism, senescence, response to endoplasmic reticulum (ER) stress, mitochondrial maintenance, oxidative stress, innate immunity, inflammation, and tumorigenesis [6–12].

MITF has been most extensively studied in melanoma, where it functions as a master regulator of melanocyte biology. The MITF-M isoform predominates in melanocytes and melanoma and governs key programs including DNA replication, repair, and cell-cycle progression [6, 13]. Genome-wide analyses of melanoma cells have confirmed that MITF target genes are enriched for pathways involved in DNA replication, repair, and mitosis, underscoring its central role in oncogenesis [14].

We recently reported that silencing MITF impairs the survival and proliferation of gastrointestinal stromal tumors (GISTs) [15]. GISTs, the most common mesenchymal sarcomas, originate from interstitial cells of Cajal (ICC), which regulate gastrointestinal tract motility, and are genetically heterogeneous [16]. Activating mutations in KIT (60–70% of cases) and PDGFRA (10–15%) are the principal, mutually exclusive oncogenic drivers [17]. Unlike melanoma, GISTs predominantly express the longest MITF isoform, MITF-A, suggesting a distinct regulatory landscape [15].

To elucidate the mechanisms by which MITF contributes to GIST biology, we performed chromatin immunoprecipitation sequencing (ChIP-seq) to map MITF-binding sites and RNA sequencing to define MITF-regulated genes. Integrated analyses revealed MITF control of genes involved in lysosome biogenesis, synaptic vesicle formation, and autophagy. Autophagy, a catabolic process that maintains cellular homeostasis, acts as a tumor suppressor in early carcinogenesis but later supports cancer cell survival and metastasis [18, 19]. In GISTs, autophagy has been linked to resistance to imatinib (IM) and other targeted therapies, highlighting autophagy-related pathways as potential therapeutic vulnerabilities [20, 21]. MITF itself has been associated with therapy resistance, and its inhibition has been shown to sensitize tumors to CDK4/6 inhibitors, reinforcing its potential as a therapeutic target [22].

Autophagy intersects with extracellular vesicle (EV) secretion, sharing common regulatory mechanisms and components, such as the mTOR pathway [23]. EVs and autophagy act together to support cellular adaptation and facilitate communication between cells. This coordination involves the identification and removal of damaged cellular components, a process managed by the endoplasmic reticulum (ER), Golgi apparatus, and lysosomes [24].

In this manuscript, we investigate how MITF regulates autophagy and extracellular vesicle (EV) biology in GIST. We show that MITF silencing differentially regulates PI3K/mTOR signaling, impairs autophagy, and significantly alters the proteomic composition of EVs without affecting their size or number. Proteomic changes include reduced KIT levels in both cells and EVs, suggesting that MITF regulates EV cargo to influence tumor signaling and invasion. Together, our findings implicate MITF as a regulator of autophagy and EV-mediated communication in GISTs, highlighting the MITF–autophagy–EV axis as a promising therapeutic target to limit tumor growth and overcome drug resistance.

## Results

### MITF target analysis in GIST cells

ChIP-seq was performed to identify genomic regions in GISTs that are potential targets for MITF. The experiments were conducted in GIST-T1 and GIST48 cells harboring clinically representative *KIT* mutations. The total number of reads and normalized tags used for peak calling are shown in Supplementary information 1 (SI1) (Tables S1 and S2). Those results are derived from the raw results based on MacS (v2.1.0), which were filtered to retain the most significant peaks (Supplementary Information 2 (SI2): SD1).

**Fig. 1:**
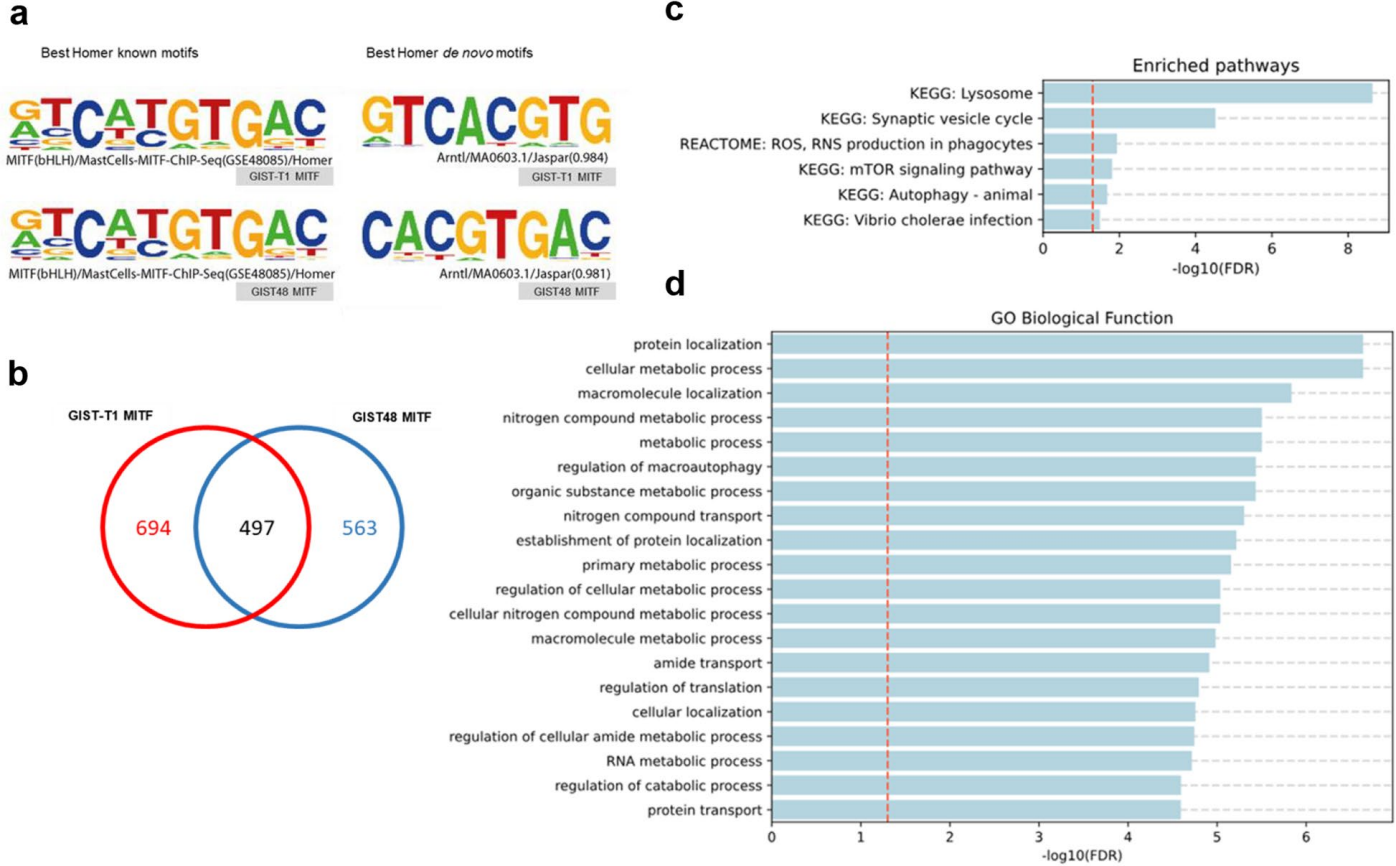
MITF ChIP-seq analysis in GIST cell lines. **a** De novo (left) and known (right) enriched motifs identified by HOMER in GIST-T1 and GIST48 MITF samples (*p* = 1e-633/1e-598 and 1e-554/1e-554), covering 66.1%/71.9% and 64.4%/67.3% of targets, respectively. **b** Venn diagram of sample-specific and shared merged regions. **c** Pathway enrichment and (**d**) GO biological functions of common ChIP-seq genes in GIST-T1 and GIST48

Known motifs were identified in GIST-T1 and GIST48 with the findMotifsGenome program of the HOMER package using default parameters and input sequences comprising ± 100 bp from the center of the top 2500 peaks. The highest ranking motif 5’-GTCATGTGAC-3’ was found in GIST-T1 and GIST48 with *p*-values of 1e-554 and 1e-484, respectively, and coverage of 68.44% in GIST-T1 and 67.31% in GIST48. Predicted de novo motifs were found with *p*-values of 1e-633 and 1e-598, respectively, and coverage of 66.09% in GIST-T1 and 71.85% in GIST48 (Fig. 1a). Moreover, the Venn diagram shows common merge regions (497) in both cell lines (Fig. 1b), thus supporting a common role for MITF downstream of *KIT* in GIST cells.

Peak analysis identified a total of 2,778 genes (SI2: SD1), with 907 common to both cell lines. The gene pathway enrichment analysis of common genes (False Discovery Rate (FDR) < 0.05) revealed KEGG (Kyoto Encyclopedia of Genes and Genomes) enrichment in processes associated with lysosomes, the synaptic vesicle cycle, mTOR signaling, and autophagy (Fig. 1c). The Gene Ontology (GO) biological function was predominant in protein location and transport, metabolic processes, and autophagy regulation (Fig. 1d). Overall, the enriched pathways—mTOR signaling, lysosomal function, protein transport, and autophagy regulation underscore key mechanisms of tumor survival and therapeutic resistance.

### Comparative MITF ChIP-seq analysis in the transcriptional regulation of GIST

To contextualize MITF within the transcriptional regulatory landscape of GIST, we compared MITF ChIP-seq peaks with publicly available datasets for known GIST transcription factors and chromatin regulators, including ETV1, a lineage-specific transcription factor in GISTs [25], HAND1, a transcriptional determinant implicated in the malignant progression of aggressive GISTs [26, 27], and the active enhancer histone mark H3K27ac [28]. MITF ChIP-seq Peaks identified in GIST-T1 and GIST48 (SI1: Table S2) were intersected with ETV1 (GSM2527316) and HAND1 (GSM2527318) ChIP-seq datasets and H3K27ac ChIP-seq peaks (GSM2527250 and GSM2527252) to evaluate MITF binding at active regulatory regions.

Our analysis revealed substantial co-localization of MITF with these transcriptional regulators. In GIST-T1 cells, 39.2% of MITF target genes overlapped with ETV1-associated genes and 22.6% with HAND1-associated genes. Moreover, 63.5% of MITF targets in GIST-T1 and 65.5% in GIST48 overlapped with H3K27ac-marked active chromatin, indicating that MITF preferentially binds active enhancer elements in both lines. Fisher’s exact tests confirmed the statistical significance of these overlaps (SI1: Fig. S1). All MITF ChIP-seq target genes overlapping ETV1, HAND1, or H3K27ac peaks, along with the relevant cell lines are listed in SI2:SD2. These findings highlight a conserved regulatory role for MITF across GIST contexts.

### MITF target genes in GIST cells

**Fig. 2:**
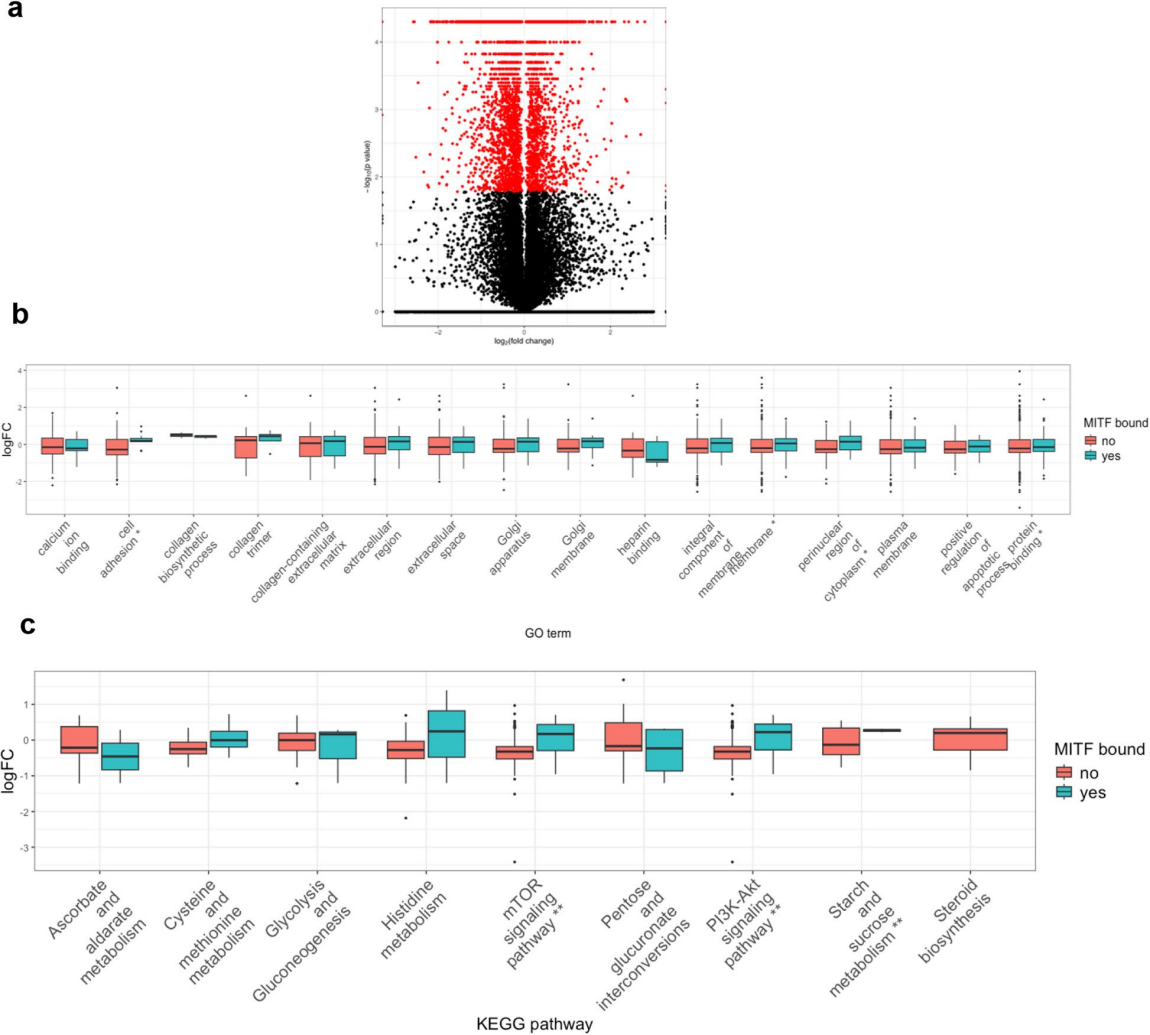
MITF-silenced transcriptomics and enrichment analysis in GIST-T1. **a** Volcano plot of log₂ fold change vs. –log₁₀ adjusted p-values; red dots indicate 5,541 DE genes (FDR < 0.05). **b** and **c** Boxplots show log fold change (logFC) distributions for MITF-bound (blue) and unbound (orange) genes across significantly enriched Gene Ontology (GO) terms and KEGG pathways. Statistical comparisons were performed with two-sided Student’s t-tests, and p-values were adjusted using the Benjamini–Hochberg method. Significance is indicated as ***p < 0.001, **p < 0.01,* *p* < 0.05; terms without asterisks are not significant

**Fig. 3:**
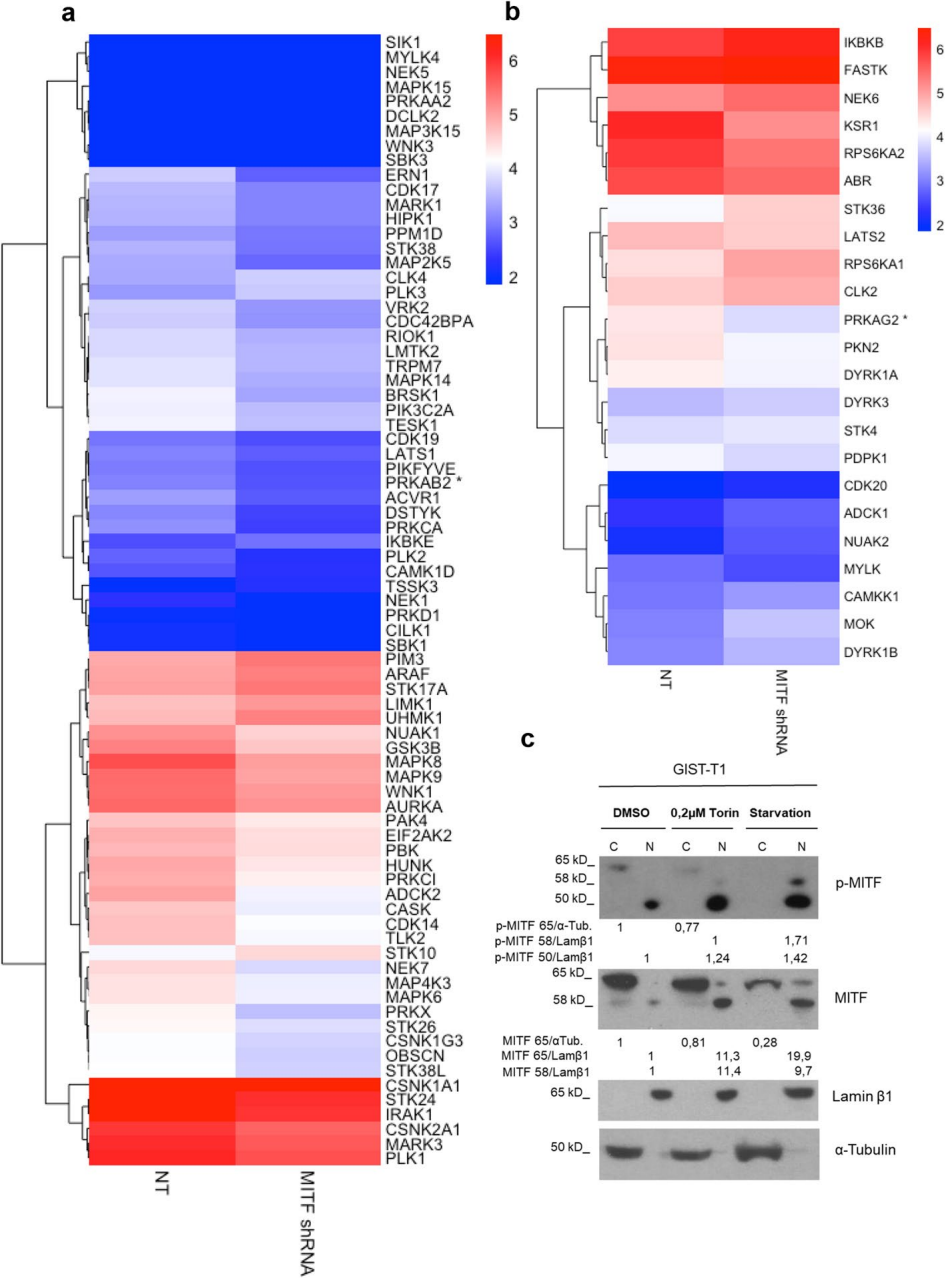
MITF-linked metabolic signaling and nuclear translocation. **a** Heatmap of MITF-unbound DEGs enriched in mTOR and PI3K–Akt pathways (|logFC|≥ 0.35). **b** Heatmap of MITF-bound DEGs in the same pathways (no logFC cutoff). Genes marked * are exclusive to mTOR; others are shared. Expression values are log₂(FPKM + 1). **c** MITF nuclear translocation after mTOR inhibition. GIST-T1 cells were treated overnight with Torin1 (0.2 µM) or starved (EBBS). Cytoplasmic and nuclear fractions were analyzed by Western blot for pMITF and MITF, with α-tubulin and Lamin β1 as loading controls. Representative blot of three experiments; protein ratios are shown

To identify potential MITF-regulated target genes, we used previously published shRNA sequences to silence MITF in GIST-T1 [15], followed by RNA sequencing. We analyzed the combined data, finding 3,345 downregulated and 2,196 upregulated genes in MITF-silenced cells compared with the non-targeting (NT) shRNA control. Among these, 136 downregulated and 147 upregulated genes overlapped with those identified in ChIP-seq experiments (SI2:SD3). The volcano plot is shown in Fig. 2a. To assess whether MITF-bound genes show distinct transcriptional regulation, we integrated RNA-seq and ChIP-seq data. Genes were classified as MITF-bound or unbound based on ChIP-seq peaks, and their log fold changes (logFC) were compared across the enriched GO terms and KEGG pathways found in the transcriptomic analysis (Figs. 2b and c). Regarding KEGG pathways, our results revealed differences in the mTOR and PI3K pathways, both of which were significantly downregulated (negative median logFC) in MITF knockdown cells. The number of differentially expressed genes (DEGs) closely matched the number of genes assayed (SI1: Table S3). Heatmap analysis revealed genes associated with the PI3K/mTOR pathway that are modulated upon MITF silencing, encompassing both indirectly regulated genes (those not directly bound by MITF (Fig. 3a) and MITF-bound targets (Fig. 3b). These findings underscore a role of MITF in orchestrating PI3K/mTOR signaling networks in GISTs.

The PI3K/AKT/mTOR pathway, frequently hyperactivated by c-KIT auto-phosphorylation, is essential for the development of GISTs and contributes to chemotherapy resistance [29, 30], critically controlling autophagy [31]. Based on ChIP-seq and RNA-seq evidence linking MITF to PI3K/mTOR signaling and autophagy, we next investigated the role of MITF in autophagy in GISTs. We induced autophagy by inhibiting mTORC1 (with Torin1) or cell starvation [32, 33]. It is known that mTORC1-mediated MITF phosphorylation promotes interaction with 14–3-3 proteins, resulting in cytoplasmic retention; mTORC1 inhibition disrupts this interaction, thereby facilitating the nuclear translocation of MITF [34]. Similarly, MITF controls cellular adaptation response to nutrient availability in an mTORC1-dependent manner [34]. Consistent with that, mTORC1 inhibition (Torin1 treatment) or nutrient starvation in GIST reduces MITF phosphorylation, enhancing its nuclear accumulation (Fig. 3c). We detected a lower molecular weight phosphorylated band in the nucleus, likely representing either a nonspecific signal or a degraded form of MITF not recognized by the specific antibody.

**Fig. 4:**
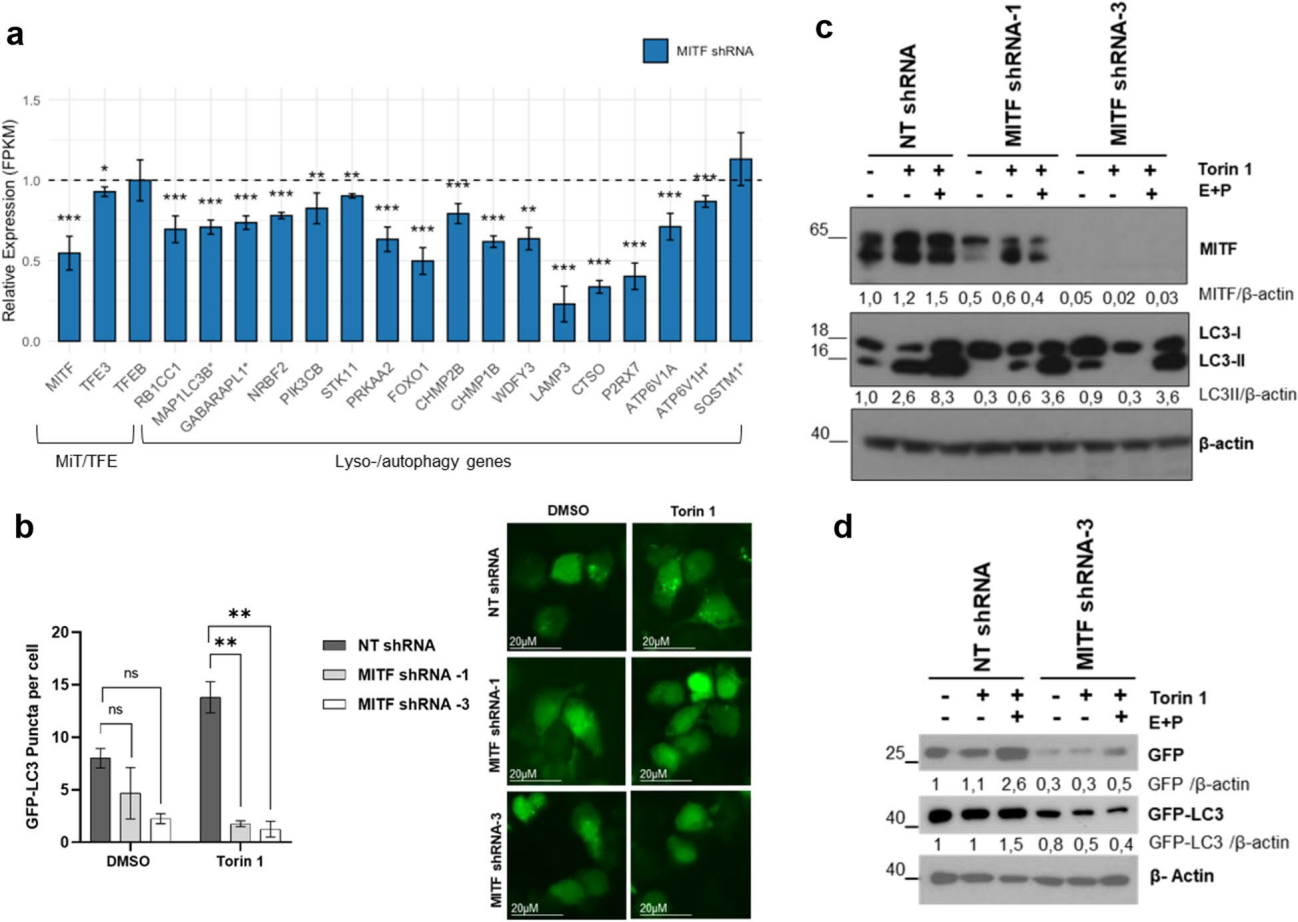
Autophagy is reduced in MITF-silenced GIST cells after Torin1 treatment. **a** Bar plots of selected gene expression (FPKM, normalized to NT controls) in MITF shRNA cells; error bars = SEM; dashed line = baseline (relative expression = 1). Significance: ****q < 0.0001, ***q < 0.001, **q < 0.01, *q < 0.05. Genes marked * are direct MITF ChIP-seq targets. **b** GFP-LC3 puncta in MITF-silenced vs. control GIST-T1 cells; ≥ 40 GFP + cells/condition analyzed. Quantification by one-way ANOVA with Bonferroni (*p < 0.05, **p < 0.01). **c** LC3II levels in NT, MITF shRNA-1, and shRNA-3 cells after Torin1 ± E64D/pepstatin; β-actin = loading control. The graph shows LC3II ratios. **d** GFP-LC3 lysosomal delivery/proteolysis assay in MITF shRNA GIST-T1 cells transfected with GFP-LC3 and treated with DMSO or Torin1 + E64D/pepstatin. Free GFP and GFP-LC3 are shown; β-actin = loading control. Representative blot of three independent experiments. Protein levels were quantified from western blots, and ratios are shown in the figures

MITF ChIP seq data revealed an enrichment in the lysosome pathway (Fig. 1b). Lysosome biogenesis is tightly coupled to autophagy, as newly formed lysosomes provide the degradative capacity required for the clearance of autophagic cargo [35]. Thus, we analyzed MITF-dependent genes involved in the lysosome-autophagy pathway. Silencing MITF in GIST-T1 cells broadly suppressed genes regulating autophagy and lysosomal function (Fig. 4a). Key initiators of autophagy, including RB1CC1 (FIP200) and PRKAA2 (AMPKα2), were reduced, suggesting impaired ULK1 activation [36, 37]. FOXO1**,** a transcription factor that drives the expression of autophagy genes, was also decreased [38]. Core components of autophagosome formation and elongation, MAP1LC3B and GABARAPL1 [39], both identified by ChIP-seq, were downregulated, indicating defective assembly. Moreover, there was decreased expression of PIK3CB, WDFY3/ALFY, and CHMP2B, which control signaling, cargo recognition, and autophagosome–lysosome fusion [40–42]. In contrast, SQSTM1/p62, which plays a role in selective autophagy and is annotated in ChIP-seq, was unaffected. Lysosomal activity was also compromised, with reduced expression of LAMP3, CTSO, and the V-ATPase subunits ATP6V1A and ATP6V1H (the latter ChIP-seq annotated), consistent with defective degradation and acidification [43, 44]. Combined, these results indicate that MITF coordinates transcriptional programs governing autophagy and lysosomal biogenesis, and MITF silencing disrupts multiple steps of these pathways. ChIP-seq tracks for MAP1LC3B, GABARAPL1, ATP6V1H, and SQSTM1 are shown in SI1: Fig. S2.

To validate these findings, we silenced MITF and monitored autophagy using green fluorescent protein (GFP)-LC3. MITF depletion markedly reduced GFP-LC3 puncta, even under basal conditions (Fig. 4b). GFP-LC3 aggregation can occur in autophagy-deficient cells [45]; therefore, we also assessed endogenous LC3-II, the lipidated form of LC3-I and a marker of autophagy. GIST cells transduced with NT or MITF shRNAs—with or without the lysosomal inhibitors E64D and pepstatin A—exhibited reduced LC3-II levels in MITF-silenced cells, correlating with MITF depletion (Fig. 4c).

To evaluate MITF’s role in autophagy flux, we used the GFP-LC3 reporter to monitor lysosomal delivery and proteolysis. In control cells, Torin1 increased free GFP—further enhanced by E64d and pepstatin A—indicating active autophagy flux, along with GFP-LC3 accumulation. In contrast, MITF-silenced cells had reduced free GFP and GFP-LC3 under the same conditions (Fig. 4d). Together with the decreased LC3 puncta, these findings indicate an early autophagy block and reinforce the role of MITF in autophagy regulation in GISTs.

We further assessed autophagy flux using the RFP-GFP-LC3 reporter, which distinguishes autophagosomes (GFP + RFP +) from autolysosomes (RFP-only) [46]. Torin1 increased both structures in control cells, but this response was markedly reduced in MITF-silenced cells (SI1: Fig. S3). Consistently, treatment with the MITF inhibitor ML329 decreased LC3-II levels in parallel with reduced MITF expression, confirming shRNA results (SI1: Fig. S4). Altogether, MITF knockdown in GIST cells revealed broad transcriptional changes, notably downregulating PI3K/mTOR signaling and key autophagy–lysosome genes. This disruption impaired autophagosome formation, lysosomal function, and overall autophagy flux.

### MITF silencing does not alter extracellular vesicle size or number in GIST cells

Autophagy and Extracellular vesicle (EV) pathways are interconnected as both rely on endolysosomal trafficking. Autophagosomes can fuse with multivesicular bodies, influencing EV cargo sorting and secretion, while EV release can serve as an alternative route when autophagy is impaired. Consequently, alterations in autophagy regulators not only disrupt autophagic flux but may also reshape EV composition and function, impacting tumor progression and cell–cell communication [24].

**Fig. 5:**
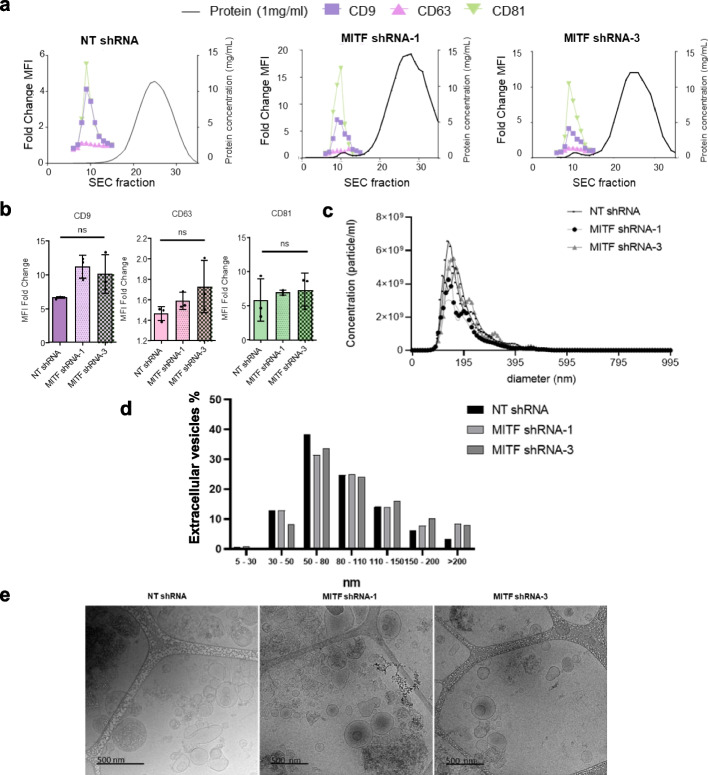
EV fraction characterization in MITF-silenced GIST cells. **a** Flow cytometry of EV markers (CD9, CD63, and CD81). **b** Fold change in mean fluorescence intensity (MFI) normalized to protein concentration (1 mg/ml). **c** Nanoparticle tracking analysis (NTA) of EV diameter and concentration. **d** Percentage of EVs from cryo-TEM samples. **e** EV size and morphology by cryo-TEM; scale bar = 500 nm

We investigated whether MITF regulates EV biogenesis or cargo in GISTs by isolating EVs from NT and MITF-silenced cells via size exclusion chromatography. EV-enriched fractions were confirmed by flow cytometry for CD9, CD63, and CD81, showing similar profiles across conditions (Fig. 5a–b). Nanoparticle tracking and cryo-EM revealed comparable EV concentrations (~ 4–6 × 10⁹ particles/mL) and size (50–150 nm), indicating that MITF silencing does not alter EV number or size (Fig. 5c-e).

**Fig. 6:**
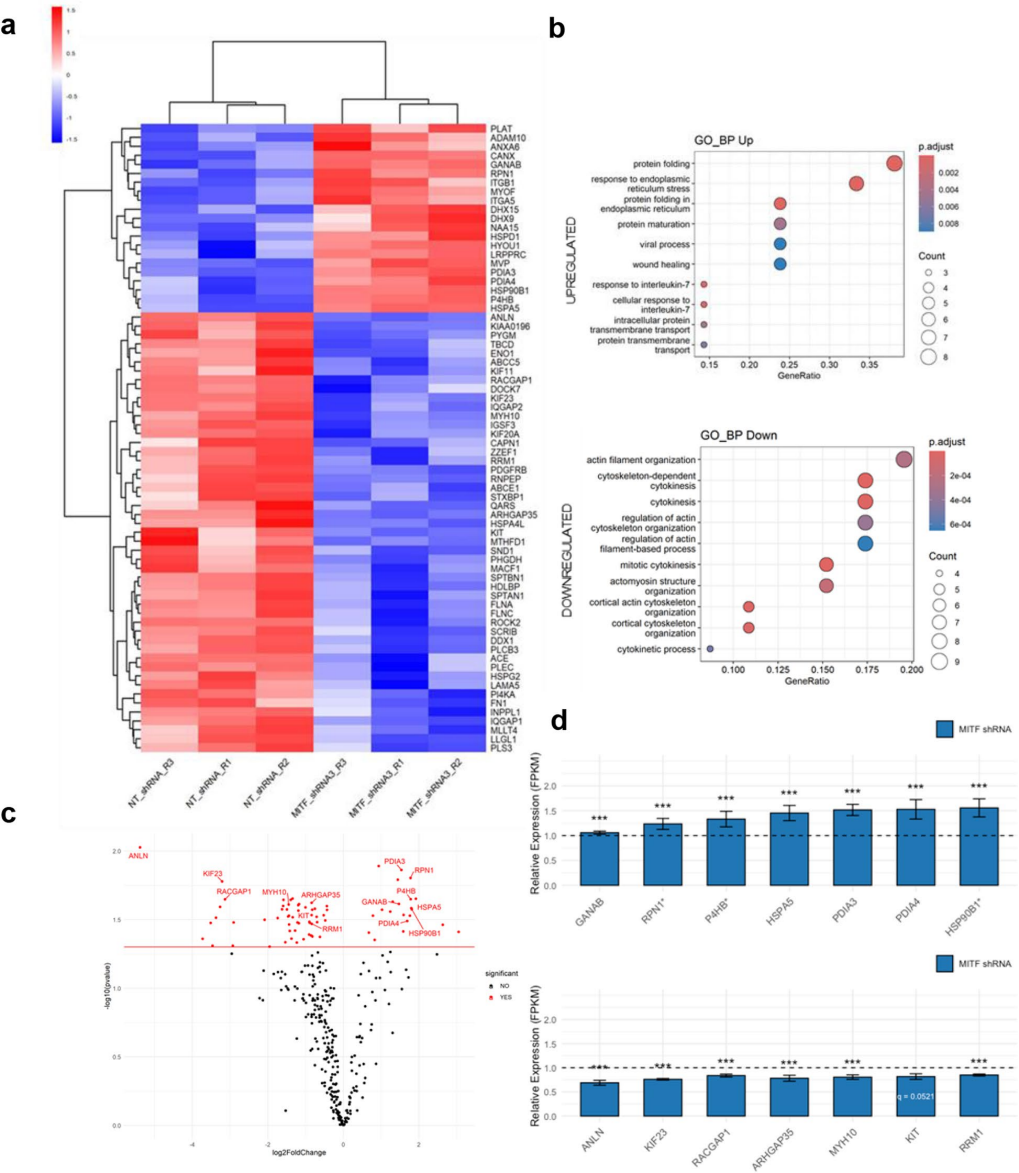
Proteomic analysis of EVs from MITF-silenced versus control GIST-T1 cells. **a** Heatmap of two-way hierarchical clustering of altered genes. **b** Over-representation analysis (ORA) of upregulated (top) and downregulated (bottom) proteins. **c** Volcano plot of selected proteins from enriched pathways. **d** Bar plots of FPKM values for selected genes in MITF shRNA vs NT controls (dashed line = 1). ER stress/protein-folding genes are upregulated; actin-cytoskeleton/cytokinesis genes are downregulated. Genes marked * are direct MITF ChIP-seq targets. Bars = mean ± SEM. Significance from Cuffdiff (BH-adjusted q-values): ****q < 0.0001, ***q < 0.001, **q < 0.01, *q < 0.05. KIT shows borderline significance (q = 0.0521). Heatmap shading: red = high, blue = low expression

We performed a proteomic analysis of EVs from MITF-silenced cells (MITFshRNA-3) (SI2: SD4). Filtering for proteins with ≥ 25 peptides, q < 0.05, and |logFC|> 0.5849, we identified 21 upregulated and 49 downregulated proteins (Fig. 6a). Over-Representation Analysis revealed enrichment of protein folding and ER stress functions among the upregulated proteins. In contrast, downregulated proteins were associated with cytokinesis, the cytoskeleton, and cell shape (Fig. 6b, SI2:SD4), consistent with MITF’s role in GIST cell division and survival [15, 47]. Gene Set Enrichment Analysis (GSEA) of MITF-silenced versus control cells (SI1: Fig. S5;SI2: SD4) revealed enrichment in cytoskeleton, cytokinesis, glycoprotein metabolism, and ER protein-folding pathways.

Heatmap analysis identified differentially expressed EV proteins from enriched pathways: ER stress/protein folding (GANAB, RPN1, P4HB, HSPA5, PDIA3, PDIA4, and HSP90B1) and cytoskeleton/cytokinesis (ANLN, KIF23, RACGAP1, ARHGAP35, MYH10, and RRM1), including KIT, the primary oncogenic driver in GISTs (Fig. 6c). Our ChIP-seq and transcriptomics data set also revealed upregulation of ER stress genes and downregulation of cytoskeleton/cytokinesis genes, several of which were bound by MITF according to ChIP-seq (Fig. 6d). KIT RNA decreased nonsignificantly, despite reduced EV protein levels.

Overall, our data show that MITF silencing in GIST cells does not change EV size or quantity but alters their protein cargo. Proteomics showed upregulation of ER stress/protein-folding proteins and downregulation of cytoskeleton and cytokinesis factors, including KIT. These data suggest MITF shapes EV composition, influencing stress responses and cell-division pathways.

### MITF silencing reduces KIT levels in GIST EVs

**Fig. 7:**
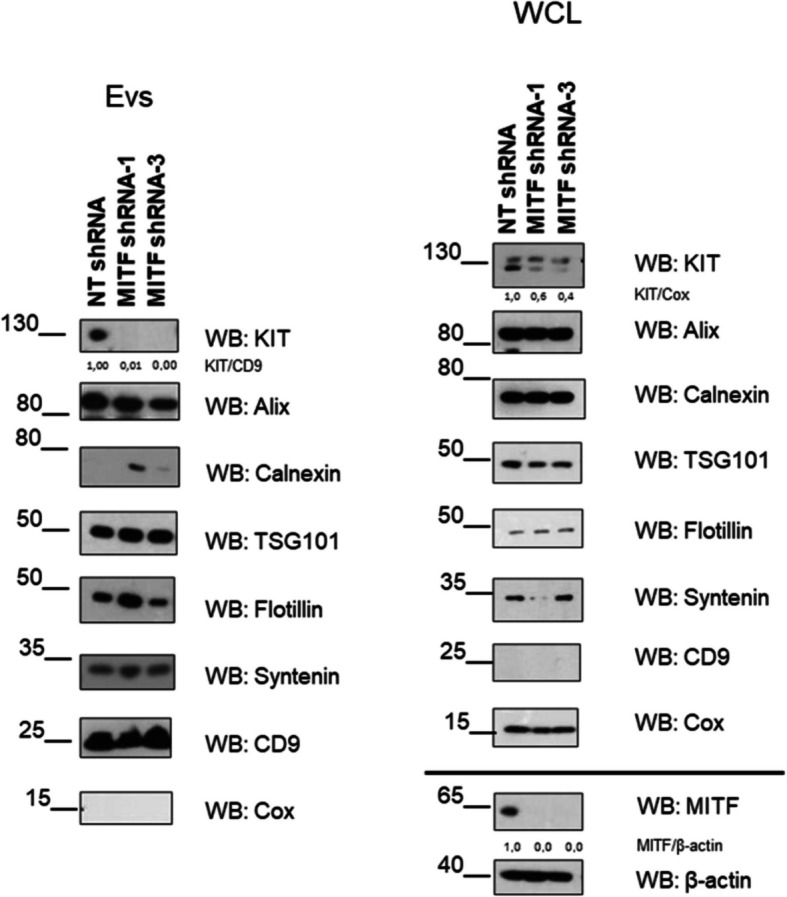
KIT protein is reduced in MITF-silenced cell lysates and exosomes. GIST-T1 cells transduced with NT shRNA, MITF shRNA-1, or shRNA-3. Lysates and exosomal fractions were analyzed by western blot for exosome markers (CD9, syntenin, flotillin, Alix, and TSG101), mitochondrial COX, the ER marker Calnexin, KIT, MITF, and β-actin. Representative blot of three independent experiments. Protein levels were quantified from western blots, and ratios are shown in the figures

Given KIT’s role as a key GIST driver, we validated EV and cell lysate levels by western blot using standard markers (Alix, Calnexin, TSG101, Flotillin, Syntenin, CD9, and Cox) according to Minimal Information for Studies of Extracellular Vesicles (MISEV) guidelines [48]. MITF silencing significantly reduced KIT in both cells and EVs (Fig. 7). As KIT-containing exosomes promote GIST invasion [49] and have therapeutic relevance [50], these results suggest MITF may modulate tumor progression via EV-mediated KIT signaling.

## Discussion

MITF has attracted considerable attention for its multifaceted role in cellular physiology, particularly in melanoma. However, growing evidence suggests that MITF may also play a critical role in the pathogenesis of GISTs. Notably, silencing or inhibiting MITF has been shown to suppress tumor growth both in vitro and in vivo [15, 47]. In this study, we investigated the role of MITF in GISTs by integrating ChIP-seq, transcriptomic, and proteomic analyses.

MITF ChIP-seq analysis, along with datasets for established GIST transcription factors (ETV1 and HAND1) [25, 26] and the H3K27ac active enhancer mark [28], revealed that MITF binds to active chromatin and enhancer regions in GIST-T1 and GIST48 cells. This co-localization and overlap suggest that MITF contributes to transcriptional activation and may functionally cooperate with key GIST transcription factors. This warrants further investigation into the role of MITF in GIST progression and therapy response.

MITF ChIP-seq in melanoma identified targets involved in DNA replication, repair, and mitosis [14]; however, in GISTs, our data indicate a distinct regulatory role. Here, MITF binds genes controlling lysosome biogenesis, mTOR signaling, and autophagy, processes central to cellular metabolism and stress adaptation. Transcriptomic analysis further supports this role, as MITF silencing significantly downregulated the mTOR and PI3K-Akt pathways, indicating that MITF sustains these oncogenic networks. This is particularly relevant for therapy, as activation of the PI3K/AKT/mTOR signaling has been associated with secondary IM resistance in GISTs [30]; IM itself has been shown to induce autophagy as a survival strategy in quiescent tumor cells [51].

Our transcriptomic analysis revealed that silencing MITF downregulates the V-ATPase subunits ATP6V1H and ATP6V1A, key components of the vacuolar H-ATPase complex required for lysosomal acidification and proper autophagic flux [52]. Concordantly, our ChIP-seq datasets identified ATP6V1H, along with core autophagy regulators such as LC3B and GABARAPL1, as direct targets of MITF (SI2:SD1 and SD3). This suggests that MITF orchestrates autophagy at multiple levels: it controls the initiation machinery through LC3B and GABARAPL1, while also ensuring lysosomal activity by transcriptionally regulating V-ATPase subunits. This dual function aligns with the lysosome-enriched pathway identified in our CHIP-seq data, together with MITF’s established role in lysosomal biogenesis [53], underscoring its importance in maintaining a functional autophagy–lysosome axis in GISTs. Significantly, disruption of this regulatory program through MITF silencing may not only compromise autophagosome formation but also impair their degradation, thereby blocking autophagic flux. Since autophagy has been implicated as a survival mechanism in GISTs, particularly under therapeutic stress (e.g., IM treatment) [51, 54], the impaired lysosomal acidification resulting from MITF loss could directly influence drug responses. This raises the possibility that MITF contributes to therapy resistance by sustaining lysosomal activity and autophagy-dependent survival. In contrast, its suppression may sensitize GIST cells to treatment by disabling this adaptive pathway.

Emerging evidence associates MITF with resistance mechanisms, particularly against CDK4/6 inhibitors. Notably, pharmacologic inhibition of MITF with ML329 restored therapeutic sensitivity, highlighting its potential as a target to counteract resistance [22].

MITF’s role in autophagy has also been observed in melanoma (SK-MEL-28) and HeLa cells [32]. Additionally, TFEB and TFE3, members of the MITF/TFE family of basic helix-loop-helix-zip transcription factors, are also associated with autophagy [55–57].

Autophagy and EVs are interconnected, jointly regulating cellular adaptation and intercellular communication. Autophagy can modulate EV secretion, while EV cargo influences autophagic activity in recipient cells. These interactions are shaped by cell type, stress, and physiological context [23, 58, 59]. Moreover, many carcinomas rely on establishing complex communication networks within and between the stroma, where EVs may play a crucial role [60].

Our study demonstrates that, while MITF silencing does not affect EV size or number, their cargo exhibits a distinct differential signature. Enhanced responses of ER stress proteins and signals related to protein folding and maturation were observed. The unfolded protein response (UPR) balances cell survival and apoptosis depending on the severity and duration of ER stress [61, 62]. Herein, the proteomic and transcriptomic analyses revealed upregulation of key ER stress and protein-folding regulators upon MITF silencing, including GANAB, RPN1, P4HB, PDIA3, PDIA4, HSPA5, and HSP90B1. Moreover, our ChIP-seq datasets identified RPN1, P4HB, and HSP90B1 as direct targets of MITF (SI2:SD1 and SD3). These proteins coordinate glycoprotein folding, N-linked glycosylation, and disulfide bond formation, ensuring proper protein maturation and UPR activation in response to stress [63, 64].

In MITF-silenced cells, EV cargo proteins, including ANLN, KIF23, RACGAP1, ARHGAP35, MYH10, and RRM1—critical regulators of cytokinesis, actin cytoskeleton organization, and DNA synthesis— are downregulated, consistent with impaired cell proliferation and viability. In parallel, cellular RNA analysis shows reduced expression of BCL2 and CDK2—key regulators of apoptosis and S-G2/M progression. This highlights that MITF supports GIST cell survival and cell-cycle progression through both intracellular transcriptional programs and EV-mediated signaling.

EVs can suppress the immune response, promote tumor progression, and serve as cancer biomarkers [65]; in melanoma, exosome cargo drives metastasis by reprogramming bone marrow progenitors [66]. Similarly, in GISTs, circulating KIT⁺ exosomes are elevated in metastatic patients, enhancing tumor metastasis and correlating with tumor burden [49, 50]. Systemic mastocytosis (SM)-derived EVs transfer functional KIT to hepatic stellate cells, activating them and promoting mastocytosis-associated liver pathology [67].

MITF impairment reduces KIT expression in both GIST and HMC-1 cells—a cellular model of mastocytosis [15, 68]. In this study, MITF knockdown consistently and significantly lowers KIT protein levels in cell lysates and EVs, potentially dampening KIT-dependent paracrine signaling. The observation that KIT mRNA levels are only modestly, not significantly, reduced upon MITF silencing, while KIT protein levels are significantly and consistently decreased (mass spectrometry and western blot), raises the possibility that the ER stress and UPR pathways enriched under MITF silencing contribute to the marked reduction or near absence of KIT receptor protein.

MITF has been identified as a potential oncogenic driver in specific sarcomas, most notably clear cell sarcoma (CCS). MITF knockdown in CCS cells results in decreased cell viability and proliferation, underscoring its crucial role in promoting tumor growth [69]. Additionally, silencing MITF in renal carcinoma cells reduces proliferation, induces cell cycle arrest at the S/G2 phases in vitro, and suppresses tumor development in vivo [70].

**Fig. 8:**
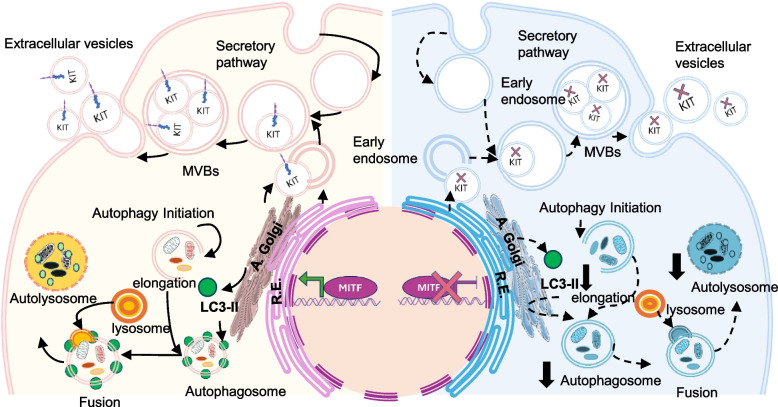
MITF silencing impairs autophagy and alters EV content in GISTs. MITF depletion reduces LC3-II levels and impairs autophagosome/autolysosome formation, highlighting MITF’s role in autophagy. The size and quantity of EVs remain unchanged; KIT expression is significantly reduced in both cells and EVs

By integrating ChIP-seq, RNA-seq, and proteomics, this study demonstrated that MITF regulates genes involved in lysosome biogenesis, vesicle formation, autophagy, and the mTOR/PI3K signaling pathway. MITF depletion disrupts the formation of autophagosomes and autolysosomes, highlighting its essential role in regulating autophagy in GISTs. Furthermore, it also alters EV composition, notably reducing KIT levels, which may influence tumor progression and metastasis (Fig. 8). These results suggest that MITF primarily regulates the composition of EV cargo rather than EV biogenesis, potentially linking autophagy-mediated lysosomal processing to selective EV packaging, a connection that warrants further investigation.

While our integrative ChIP-seq, RNA-seq, and exosome proteomics approach provides a broad view of MITF-regulated pathways in GIST, several limitations remain. Findings are based on two cell lines, and transcript changes may not reflect protein levels. Exosome proteomics captures only a subset of proteins; thus, an in vivo or additional model validation is needed to confirm the functional relevance.

## Conclusions

This study highlights MITF as a promising target for therapeutic intervention in GISTs owing to its regulatory influence on key pathways involved in tumor growth, autophagy, and metastasis. Further exploration of the molecular mechanisms of MITF and its interaction with downstream effectors could provide valuable insights into GIST pathobiology and guide the development of novel therapeutic strategies targeting MITF.

## Material and methods

### Cells, antibodies, and reagents

Human GIST cell lines were provided by Dr. S. Bauer (University Duisburg-Essen, Germany). IM-resistant GIST48 cells (p.Asp820Ala; p.Val560Asp, RRID: CVCL_7041) [71] were cultured in Ham’s F-10 medium (Lonza, Basel, Switzerland) with 15% foetal bovine serum, 1% L-glutamine, 50 U/ml penicillin/streptomycin, 30 mg/ml bovine pituitary extract, and 0.5% MITO + Serum Extender (Fisher Scientific, Pittsburgh, PA). IM-sensitive GIST-T1 (KIT p.Val560_Tyr578del, RRID: CVCL_4976) cells were described previously [72]. All cell lines were routinely tested for Mycoplasma.

Antibodies: mouse anti-KIT (Ab81), anti-CD81 (G0709), rabbit anti-GFP (Santa Cruz Biotechnology, Santa Cruz, CA, USA); anti-MITF (D5G7V), anti-Alix, anti-CD9 (13,174), anti-LC3 I/II (81,631) (Cell Signaling, Danvers, MA, USA); anti-p62/SQSTM1 (MBL, Nagoya, Japan); mouse anti-COX IV (A-21347, Invitrogen, Waltham, MA, USA); mouse anti-β-actin (AC-40, Sigma, St. Louis, MO, USA); anti-syntenin (ab8133267), anti-TSG101 (ab30871), anti-CD63 (ab8219), anti-calnexin (ab22595) (Abcam, Cambridge, UK); anti-mouse and anti-rabbit IgG peroxidase (DAKO, Carpinteria, CA, USA and BioRad Hercules, CA, USA); and flotillin1 anti-mouse (#610,820, BD Biosciences, San Diego, CA, USA). ML329 was purchased from Axon Med Chem (Groningen, Netherlands).

### Chromatin Immunoprecipitation Sequencing (ChIP-seq)

GIST-T1 and GIST48 cells were frozen at −120 °C and processed by Active Motif (Carlsbad, CA) for ChIP-seq (RRID: SCR_001237). Cells were cross-linked with 1% formaldehyde, quenched with glycine, lysed, and chromatin was fragmented (300–500 bp) using an EpiShear sonicator (cat# 53,051). Input DNA was prepared by RNase/proteinase K treatment, heat reversal, SPRI bead cleanup, and quantification. Chromatin yield was calculated from the starting volume.

For ChIP, 30 µg of chromatin was precleared with protein G agarose beads (Invitrogen) and immunoprecipitated with 5 µg anti-MITF antibody (Active Motif, cat# 39,789, lot# 11,313,002). Complexes were washed, eluted, treated with RNase/proteinase K, and cross-links reversed overnight at 65 °C. DNA was purified by phenol–chloroform extraction and ethanol precipitation. Enrichment at selected loci was validated by quantitative PCR (qPCR) in triplicate using SYBR Green Supermix (Bio-Rad), normalized to Input.

ChIP and Input DNAs were converted into sequencing libraries using automated enzymatic processing (Apollo 342, Wafergen/Takara), PCR-amplified, quantified, and sequenced on an Illumina NextSeq 500 (75 nt, single-end).

### Bioinformatic analysis: CHIP-seq

FASTQ files were aligned to the human genome (hg38) using BWA (RRID: SCR_010910) with the aln/samse algorithm and default settings. Only Illumina-passed reads with ≤ 2 mismatches, unique alignment, and mapping quality ≥ 25 were retained; duplicates were removed. Alignments were extended in silico to 200 bp and assigned to 32-nt bins, generating genomic signal maps stored as bigWig files. Peaks were identified with MACS2 (v2.1.0) at *p* < 1e-7, excluding ENCODE blacklist and false-positive regions. Active Motif’s proprietary pipeline integrated signal maps and peak calls for downstream analysis.

Motif enrichment was assessed with HOMER (RRID: SCR_010881) using ± 200 bp regions around the top 2,500 MACS2 peaks. Functional enrichment of associated genes was performed using GO, KEGG pathways (RRID: SCR_012773), and UniProt keywords.

### Transcriptomic analysis

Total RNA was extracted in triplicate from GIST-T1 cells transduced with MITF shRNA-1, MITF shRNA-3, or non-targeting (NT) shRNA control, and shipped at –80 °C to Macrogen for RNA-seq. Genomic DNA was removed using DNase treatment. For library preparation, poly(A)-purified mRNA was isolated for coding transcripts, while ribo-zero depletion was used for non-coding RNAs (e.g., lincRNAs). Purified RNAs were randomly fragmented, reverse-transcribed into cDNA, and ligated to sequencing adapters. Following PCR amplification, libraries with insert sizes of 200–400 bp were selected. Final libraries were sequenced on an Illumina platform using paired-end reads.

### Bioinformatic analysis: RNA seq

Read quality was assessed by calculating base composition, GC content, and overall statistics. Adapter sequences, low-quality reads, contaminants, and PCR duplicates were removed. Cleaned reads were aligned to the reference genome with HISAT2 (RRID: SCR_015530), a splice-aware aligner. Transcript assembly was performed using StringTie, and expression levels were quantified as raw read counts and normalized values. Normalization was calculated based on transcript length and sequencing depth, reported as FPKM (Fragments Per Kilobase of transcript per Million Mapped reads)/RPKM (Reads Per Kilobase of transcript per Million mapped reads) and TPM (Transcripts Per Kilobase Million).

#### Preprocessing

Quality control was performed on Fastq libraries using FastQC (RRID: SCR_014583) [73]. Subsequently, Fastq files were preprocessed using Cutadapt 1.18 [74] and Prinseq-lite-020.4 [75] to eliminate primers and low-quality sequences.

#### Mapping and differential expression analyses

Preprocessed Fastq files were mapped on the human genome GRCh38 version provided by the Ensembl release 108 [76], using TopHat 2.1.1 (RRID: SCR_013035) [77]. The overall read mapping rates successfully ranged from 87 to 92% of reads mapped. Bam files resulting from mapping with TopHat were used as input to Cufflinks/Cuffdiff (RRID: SCR_014597/RRID: SCR_001647) to perform a differential expression (DE) analysis between the MITF-silenced group (six samples) and the control group (three samples) as described elsewhere [78]. DEGS supported by a Benjamini–Hochberg FDR correction < 0.05 were considered statistically significant. Statistical and graphical analyses were performed using CummeRbund (RRID: SCR_014568) [79].

All DEGs were provided with annotations (regarding gene names, descriptions, GO, enzyme commission (EC) numbers, and metabolic pathways) using Biomart (RRID: SCR_019214), Ensembl (RRID: SCR_002344) [76], and the KEGG [80] knowledge database. Finally, an enrichment analysis of GOs and metabolic pathways was performed using GOseq [81] based on the differential expression results. To perform these analyses, all assayed genes were previously provided with annotations of GOs and metabolic pathways retrieved from Ensembl and Biomart [76, 82] and the KEGG database [80], respectively. Enriched GO terms and metabolic pathways supported by an FDR-corrected *p*-value < 0.05 in the resulting Wallenius and/or Sampling distribution were considered statistically significant. All RNAseq data processing, differential expression, and enrichment analysis were executed using the TopHat-Cufflinks pipeline provided by the RNASeq app v2.2.10 of the GPRO suite [83].

#### Co-binding analysis between MITF and other transcription factors or chromatin marks

Publicly available ChIP-seq datasets were downloaded from the Gene Expression Omnibus (GEO) [84]. Specifically: ETV1 in GIST-T1 (GSM2527316), HAND1 in GIST-T1 (GSM2527318), H3K27ac in GIST-T1 (GSM2527250), and H3K27ac in GIST48 (GSM2527252). These datasets were originally aligned to the hg19 version of the human genome. To harmonize coordinates with our MITF ChIP-seq data (generated on hg38), peak coordinates were converted to hg38 using UCSC liftOver [85].

For each factor, we annotated peaks to genes using BEDTools [86], assigning them if they overlapped gene bodies or promoter regions. Gene symbols and coordinates were obtained from the Ensembl gene annotation (release 108, GRCh38) [76]. To define a consistent gene universe, we extracted unique gene symbols from this annotation, resulting in a total of 41,129 genes after eliminating redundancy.

Gene sets associated with MITF and each factor were compared to quantify overlap. The percentage of MITF target genes shared with each factor was visualized using a barplot.

To statistically assess the significance of co-binding, we performed Fisher’s exact tests using the defined universe of 41,129 unique gene symbols. Contingency tables were constructed for each comparison based on the number of shared and exclusive genes between MITF and the other factor. Odds ratios and *p*-values were calculated using the Fisher_exact function from the scipy.stats package [87] in Python. All outcomes are presented in SI1: Fig. S1 and detailed in SI2:SD2.

To visually represent co-binding events, we generated genome-wide ChIP-seq tracks for MITF, ETV1, HAND1, and H3K27ac in GIST-T1 and GIST48 cells using the Integrative Genomics Viewer (IGV) [88]. Two tracks are included for each dataset: one showing all called peaks (blue) and another highlighting those overlapping with MITF peaks (red). The ChIP-seq tracks displayed were obtained after comparing against the Input as the negative control. These genome-wide visualizations, which provide an overview of co-occupancy patterns and potential interactions, are shown in SI1: Fig. S1.

In addition, we focused on four key autophagy-related genes (*MAP1LC3B*, *GABARAPL1*, *ATP6V1H*, and *SQSTM1*) to illustrate co-binding at specific loci. IGV tracks for each dataset, along with tracks showing overlapping peaks with MITF, were generated for both GIST-T1 and GIST48 cells. These views are presented in SI1: Fig. S2.

### Bioinformatic analysis: proteomics

Raw files were processed with MaxQuant (RRID: SCR_014485) [89] v1.6.17.0 using the integrated Andromeda Search engine [90]. All data were searched against a target/decoy version of the human Uniprot Reference Proteome (RRID: SCR_002380). The first search peptide tolerance was set to 20 ppm, and the main tolerance was set to 4.5 ppm. Fragment mass tolerance was set to 20 ppm. Trypsin was specified as the enzyme, cleaving after all lysine and arginine residues and allowing up to two missed cleavages. Carbamidomethylation of cysteine was specified as a fixed modification, and peptide N-terminal acetylation, methionine oxidation, asparagine deamidation, and glutamine and pyro-glutamate formation from glutamine and glutamate were considered variable modifications with a total of two per peptide. “Maximum peptide mass” was set to 7500 Da, the “modified peptide minimum score” and “unmodified peptide minimum score” were set to 25, and the rest was set to default values, including the FDR limit of 1% on both the peptide and protein levels. The obtained quantitative data were exported to Perseus software (version 1.6.15.0) [91] for statistical analysis and data visualization. An unpaired Student’s t-test was used to compare groups directly for total protein analysis. Statistical significance was set at *p* < 0.05 in all cases, and a 1% FDR threshold was considered.

Differentially expressed proteins were considered significant when their LogFC was ≤ −0.38 (downregulated proteins) or ≥ 0.38 (upregulated proteins).

GSEA for all annotated proteins ranked by their t-test statistic was conducted using the gseGO and gseKEGG functions of the clusterProfiler (RRID: SCR_016884) R package (v.4.8.3) [92]. Data visualization of the clustered top GSEA terms was performed using the treeplot function in the enrichplot R package (v. 1.20.3) [93].

Afterward, a more restrictive filter was performed. Proteins represented by at least 25 peptides, a q-value < 0.05, and logFC ± 0.5849 were filtered and described in a heatmap using the heatmap R package (v.1.0.12) [94, 95]. ORA analysis on upregulated and downregulated proteins was performed using enrichGO and enrichKEGG of the clusterProfiler (v.4.8.3) R package. ORA analysis results are represented by the dot plot function from the enrichplot (v. 1.20.3) R package.

## Supplementary Information

Supplementary Material 1.

Supplementary Material 2.

## Data Availability

Data are available in a public, open-access repository 10.1101/2024.09.10.612253v1, doi: 10.1101/2024.09.10.612253. Raw data have been deposited in the NCBI SRA archive with BioProject RRID: SCR_004801 PRJNA748541 and BioSample records (SAMN20336602, SAMN20336603, SAMN20336604, and SAMN20336605). The manuscript has data included as electronic supplementary material.
