# Supplementary material for: MITF regulates autophagy and extracellular vesicle cargo in gastrointestinal stromal tumors
Source: Mol Biomed. 2025 Oct 31;6:92. doi: 10.1186/s43556-025-00329-9 (PMC12575911; doi:10.1186/s43556-025-00329-9)
Supplement: Supplementary file 1 — Supplementary Material 1. [file 43556_2025_329_MOESM1_ESM.pdf]

Supplementary Information

Supplementary Material and Methods

Supplementary Tables

Supplementary Figures

MITF Regulates Autophagy and Extracellular Vesicle Cargo in Gastrointestinal Stromal Tumors

Elizabeth Proaño-Pérez[1,2,3,4], Eva Serrano-Candelas[1,5], Mario Guerrero[1], David Gómez-Peregrina[6], Carlos Llorens[7], Beatriz Soriano[7], Ana Gámez-Valero[1], Marina Herrero-Lorenzo[1], Eulalia Martí[1], César Serrano[6,8], Margarita Martin[1,2]

[1] Biochemistry and Molecular Biology Unit, Biomedicine Department, Faculty of Medicine and Health Sciences, University of Barcelona, Barcelona 08036, Spain

[2] Multidisciplinary and translational research in inflammation and immunoallergy (METRI² A), Institut d'Investigacions Biomediques August Pi i Sunyer (IDIBAPS). Barcelona 08036, Spain

[3] Facultad de Ciencias de la Salud, Universidad Técnica de Ambato, 180105, Ambato-Ecuador

[4] Nutrigenx. Facultad de Ciencias de la Salud, Universidad Técnica de Ambato, 180105, Ambato-Ecuador

[5] ProtoQSAR SL, Centro Europeo de Empresas Innovadoras (CEEI), Parque Tecnológico de Valencia, Paterna 46980, Valencia. Spain.

[6] Sarcoma Translational Research Program, Vall d'Hebron Institute of Oncology (VHIO), Hospital Universitario Vall d'Hebron, Vall d'Hebron Barcelona Hospital Campus, C/ Natzaret, 115-117, 08035, Barcelona, Spain.

[7] Biotechvana, Parque Científico Universidad de València, Paterna 46980, Valencia. Spain.

[8] Department of Medical Oncology, Vall d'Hebron University Hospital, Barcelona 08035, Spain.

SUPPLEMENTARY MATERIAL AND METHODS

Gene overexpression or MITF silencing in GIST

pEGFP-LC3 and dual-fluorescence mRFP-GFP-LC3 plasmids were gifts from Tamotsu Yoshimori (plasmid # 21073 and # 21074, Addgene, Watertown, MA, USA) [1,2]. GFP plasmids were transfected into GIST-T1 cells using Lipofectamine LTX (Invitrogen) according to the manufacturer's instructions, with slight modifications. Relation (2,5 µg DNA +13,75 µL lipofectamine in a final volume of 2mL). Lentiviral particles to silence the MITF gene expression (MITF shRNA-1 and MITF shRNA-3) were previously described elsewhere [3]. Infected cells were cultured with puromycin (1µg/ml). NT shRNA or MITF shRNA GIST-T1 cells were transiently transfected with pEGFP-LC3. Dual-fluorescence mRFP-GFP-LC3 stable transfectants were produced using Lipofectamine LTX, as described above, and selected with G481 (400µg/ml) for 4 weeks. Dual-fluorescence mRFP-GFP-LC3GFP stable transfectants were infected with NT shRNA or MITF shRNA and maintained in medium containing puromycin (1 µg/ml) and G418 (400 µg/ml).

Western blotting

Cellular fractioning was performed as described elsewhere [4]. Western blotting was conducted as described [5]. According to the manufacturer's recommendations, the total protein concentrations were determined using the Protein Assay Dye Bio-Rad Kit (Bio-Rad Laboratories (RRID: SCR_008426), Inc., Hercules, CA, USA). Electrophoresis and protein blotting were performed using NuPageTM 4–12% and 12% Bis-Tris Gel 1.5 mm * 15 w (Invitrogen, Waltham, MA, USA); after that, the proteins were electrotransferred to polyvinylidene difluoride (PVDF) membranes (Millipore, Bedford, MA, USA). Membranes were blocked and incubated with primary antibodies. After TTBS washes, membranes were incubated with secondary anti-mouse or anti-rabbit antibodies. Proteins were visualized by enhanced chemiluminescence (Western BrightTM ECL, Advansta, San Jose, CA, USA).

Autophagy experiments

Cells were starved overnight with buffer EBSS (B32750, R&D Systems, Minneapolis, MN, USA) or treated for 16h with mTOR inhibitor torin1 (200 nM)(Sigma Aldrich, St Louis, MO, USA) and protease inhibitors, pepstatin A (10 µg/mL), E64D (10 µg/mL), and chloroquine (10 µM) (Sigma-Aldrich). Autophagy induction was performed in GIST-T1 after silencing or inhibiting MITF with ML329 (5 µM).

MITF silencing

GIST-T1 were transduced with NT shRNA, MITF shRNA-1, or MITF shRNA-3 lentiviral particles and maintained with puromycin 1ug/mL to select transduced cells. On the 5th day post-transduction, cells were transfected with pEGFP-LC3 and divided for protein expression analysis in a 6-well plate ($1x10^6$ cells/well) and resuspended ($0.1x10^6$ cells/well) in a 24-well plate on cover slides. After that, at six days post-transduction, cells in both well-plates were treated with torin1 (200nM) +/- pepstatin A (10ug/mL) and E64D (10ug/mL) for 16h. Cells that grew on coverslips were fixed, and GFP-LC3 expression was checked under the microscope. Cells that grew in the p6-plate were lysed for Western blot assays.

An RFP-GFP-LC3 GIST-T1 stable cell line was obtained to analyze autophagy flux. The expression of GFP and RFP was monitored for four weeks. Cells were transduced with NT shRNA or MITF shRNA-1, -3 and maintained with puromycin (1µg/ml) and G418 (400 µg/ml). On the 5th day, cells were harvested and divided for protein expression analysis in a conventional 6-well plate ($1x10^6$ cells/w) and resuspended ($0.1x10^6$ cells/w) in a 24-well plate on cover slides for immunofluorescence studies at six days cells were treated with torin 1 (200nM) +/- pepstatin A 10µg/mL, and E64D 10µg/mL for 16h. Cells that grew on coverslips were fixed, and cells that grew in a 6-well plate were recollected in a lysis buffer to analyze protein concentration and perform a Western blot.

Quantitative GFP-LC3 and RFP-GFP-LC3 analyses.

GFP or dual RFP-GFP expression was measured by fluorescence microscopy. For this, cells were fixed with 4% paraformaldehyde (Merck KGaA, Darmstadt, Germany), and nuclei were stained with Hoechst 3342 (Merck KGaA). They were

then mounted on a slide using the mountant liquid ProLong Gold Antifade Mountant (Thermo Fisher Scientific). Samples were observed with epi-fluorescent microscopy (Leica AF600) with 60x magnification.

Dots were quantified in GFP-LC3-transfected or RFP-GFP-LC3 stable GIST-T1 cells. A minimum of five dots per cell in GFP-LC3 or RFP-GFP-LC3 stable GIST-T1 was needed to be considered a positive cell. Regarding GFP-LC3-transfected cells, at least 150 GFP-positive cells per condition were analyzed, and results were expressed as a percentage of GFP-LC3 dot-positive cells/the total number of transfected cells. For RFP-GFP-LC3 tests, at least 30 RFP-GFP-positive GIST-T1 cells for each experimental condition were analyzed. Autophagosomes showed both RFP and GFP signals, while autolysosomes were defined as RFP-positive dots [6]. The number of autolysosomes was calculated as follows: Autolysosomes = RFP/positive dots – GFP/positive dots.

Isolation of extracellular vesicles from culture media

Extracellular vesicle isolation and characterization

Extracellular vesicles (EVs) were isolated from the culture media of lentiviral transduced NT shRNA or MITF shRNA-3 GIST-T1 by size-exclusion chromatography (SEC) as previously described protocol [7]. Briefly, cells were cultured in DMDM supplemented with 10% FBS. On the 4th day after lentiviral transduction, this media was replaced with DMDM and supplemented with EV-depleted FBS. EV-depleted FBS was obtained through ultracentrifugation at 4ºC, 100.000 g for 18 h. After 72 h of culture, conditioned media (CM) were collected and subsequently centrifuged at 500 g, 4ºC for 5 min and at 2000g, 4ºC for 15 min. This CM was further concentrated using Amicon Ultra-15 100-kDa ultrafilter (UF) units (Millipore, #UFC910024), obtaining 2 mL of concentrated conditioned media (CCM) that was stored until further use. In parallel, the remaining cells were counted, and viability was checked using trypan blue staining. Cells were lysed and stored at -80ºC.

EVs were isolated from the CCM using size-exclusion chromatography (SEC), as reported elsewhere [7,8]. Sepharose-CL2B (Sigma Aldrich, St Louis, MO, USA) was stacked in a puriflash column Dry Load Empty 12g (20/pk) from Interchim (France)-Cromlab, S.L. (Barcelona, Spain). 500 µl/tube fractions were recollected

(34 tubes, 1.5 mL) using PBS1x as elution buffer. Fractions obtained after SEC were analyzed for protein content by measuring their absorbance at 280 nm in a Thermo Scientific Nanodrop® ND-100 (Thermo Fisher Scientific, Waltham, MA). The SEC fractions showing a low/minimal protein concentration were selected for the forthcoming EV analyses using classical EV-associated markers CD9, CD63, and CD81 by bead-based flow cytometry assay as previously reported [8,9]. The presence of specific EV-markers CD9, CD81, and CD63 was determined in these fractions by flow cytometry (FACSDiva (RRID: SCR_001456) Version 6.1.3, BD Biosciences, New Jersey, USA). Mean fluorescence intensity (MFI) values were plotted (Flow Jo software (RRID: SCR_008520), Tree Star, Ashland, OR), and tetraspanin-positive fractions with the highest MFI (fractions 7–12 from our SEC column) were considered EV-enriched fractions and pooled for the subsequent analysis.

EV-enriched pools were characterized by nanoparticle tracking analysis (NTA) and cryo-electron microscopy (cryo-EM), which obtained a rough concentration and checked for morphology and size. Samples were analyzed at the Services Technical Scientific of the Universitat Autonoma de Barcelona.

The size distribution of EVs was further quantified by measuring the diameter of vesicles in each condition using ImageJ (RRID: SCR_003070).

Label-free quantitative proteomics

Purified extracellular vesicles were resuspended in Lysis Buffer containing 7M urea, 2 M thiourea, and 50 mM DTT. Protein extracts were diluted in a Laemmli sample buffer and loaded into a 1,5 mm thick polyacrylamide gel with a 4% stacking gel cast over a 12.5% resolving gel. The run was stopped when the front entered 3 mm into the resolving gel so that the whole proteome became concentrated in the stacking/resolving gel interface. Bands were stained with Coomassie Brilliant Blue, excised from the gel, and protein enzymatic cleavage was carried out with trypsin (Promega; 1:20, w/w) at 37 °C for 16 h as previously described [10]. Purification and concentration of peptides were performed using C18 Zip Tip Solid Phase Extraction (Millipore).

Liquid chromatography-mass spectrometry (LC-MS/MS)

Peptide mixtures were separated by reverse phase chromatography using an UltiMate 3000 UHLPC System (Thermo Scientific) fitted with an Aurora-packed emitter column (Ionopticks, 25 cm x 75 µm ID, 1.6 µm C18). Samples were first loaded for desalting and concentration into an Acclaim PepMap column (ThermoFisher, 0,5 cm x 300 µm ID, 5 µm C18) packed with the same chemistry as the separating column. Mobile phases were 100% water, 0.1% formic acid (FA) (buffer A), and 100% Acetonitrile 0.1% FA (buffer B). Column gradient was developed in a 120-minute, two-step gradient from 5% B to 20% B in 90 minutes and 20%B to 32% B in 30 minutes. The column was equilibrated in 95% B for 10 minutes and 5% B for 20 minutes. During all processes, the precolumn was in line with the column, and flow was maintained all along the gradient at 300 nl/min. The column temperature was maintained at 40 °C using an integrated column oven (PRSO-V2, Sonation, Biberach, Germany) and interfaced online with the Orbitrap Exploris 480 MS. The spray voltage was set to 2 kV, the funnel RF level was at 40, and the heated capillary temperature was set at 300 °C. For DDA experiments, full MS resolutions were set to 1200,000 at m/z 200, and the entire MS AGC target was set to Standard with an IT mode Auto. The mass range was set to 375–1500. The AGC target value for fragment spectra was set to Standard, with a resolution of 15,000 and a cycle time of 3 seconds. The intensity threshold was kept at 8E3. Isolation width was set at 1.4 m/z. The normalized collision energy was set at 30%. All data were acquired in centroid mode using positive polarity. The peptide match was set to off, and isotope exclusion was enabled.

EVs Protein Extraction and Western Blot Analysis

Western blot was used to assess EV and non-EV markers in the EV pools. For this purpose, the protein concentration of EVs was measured using the Micro BCA Protein Assay Kit (Thermo Fisher Scientific). An equal amount of each sample (20 µg/25 µL) was mixed with reducing 5X Pierce Lane Marker Reducing Sample Buffer (Thermo Fisher Scientific), boiled for 5 min at 95 °C, and subjected to Western blot as mentioned above.

[1] Kabeya Y. LC3, a mammalian homologue of yeast Apg8p, is localized in autophagosome membranes after processing. The EMBO Journal 2000;19:5720–8. https://doi.org/10.1093/emboj/19.21.5720.

[2] Kimura S, Noda T, Yoshimori T. Dissection of the Autophagosome Maturation Process by a Novel Reporter Protein, Tandem Fluorescent-Tagged LC3. Autophagy 2007;3:452–60. https://doi.org/10.4161/auto.4451.

[3] Proaño-Pérez E, Serrano-Candelas E, García-Valverde A, Rosell J, Gómez-Peregrina D, Navinés-Ferrer A, et al. The microphthalmia-associated transcription factor is involved in gastrointestinal stromal tumor growth. Cancer Gene Therapy 2023;30:115–7. https://doi.org/10.1038/s41417-022-00539-1.

[4] Suzuki K, Bose P, Leong-Quong RY, Fujita DJ, Riabowol K. REAP: A two minute cell fractionation method. BMC Research Notes 2010;3:294. https://doi.org/10.1186/1756-0500-3-294.

[5] Serrano-Candelas E, Ainsua-Enrich E, Navinés-Ferrer A, Rodrigues P, García-Valverde A, Bazzocco S, et al. Silencing of adaptor protein SH3BP2 reduces KIT/PDGFRA receptors expression and impairs gastrointestinal stromal tumors growth. Molecular Oncology 2018;12:1383–97. https://doi.org/10.1002/1878-0261.12332.

[6] Ozturk DG, Kocak M, Akcay A, Kinoglu K, Kara E, Buyuk Y, et al. MITF-MIR211 axis is a novel autophagy amplifier system during cellular stress. Autophagy 2019;15:375–90. https://doi.org/10.1080/15548627.2018.1531197.

[7] Monguió-Tortajada M, Gálvez-Montón C, Bayes-Genis A, Roura S, Borràs FE. Extracellular vesicle isolation methods: rising impact of size-exclusion chromatography. Cellular and Molecular Life Sciences 2019. https://doi.org/10.1007/s00018-019-03071-y.

[8] Gámez-Valero A, Monguió-Tortajada M, Carreras-Planella L, Franquesa M, Beyer K, Borràs FE. Size-Exclusion Chromatography-based isolation minimally alters Extracellular Vesicles' characteristics compared to precipitating agents 2016;6:1–9.

[9] Monguió-Tortajada M, Morón-Font M, Gámez-Valero A, Carreras-Planella L, Borràs FE, Franquesa M. Extracellular-Vesicle Isolation from Different Biological Fluids by Size-Exclusion Chromatography. Current Protocols in Stem Cell Biology 2019;49. https://doi.org/10.1002/CPSC.82.

[10] Shevchenko A, Tomas H, Havli J, Olsen J V., Mann M. In-gel digestion for mass spectrometric characterization of proteins and proteomes. Nature Protocols 2006;1:2856–60. https://doi.org/10.1038/nprot.2006.468.

Supplementary Table 1.- Experimental ChIPseq results and Tags used for peak calling.

Sample	GIST-T1_MITF	GIST-48_MITF	GIST-T1	GIST-48
Total number of reads	41471147	46305582	42506632	41367319
Total number of alignments (hg38)	38653529	41363994	41043763	39464884
Unique alignments (-q 25)	34627501	36407590	36257041	34215416
Unique alignments (without duplicate reads)	12849635	14858457	34828785	32994059
Usable number of Tags	12831569	14843347	34807357	32972647
Normalized tags	12831569	12831569	12831569	12831569
ChIP tags used for peak calling	12831569	12831569	12831569	12831569
Input tags used for peak calling	12831569	12831569		

Supplementary Table 2- Peak calling and obtained FRIP

Features	GIST-T1_MITF	GIST-48_MITF
Paired peaks	51863	29807
Predicted fragment length	200	204
Final MACS peaks	1194	1062
ENCODE blacklisted	2	1
Filtered peaks	1192	1061
FRIP (in percent)	0.41	0.31

Supplementary Table 3. Differentially enriched Metabolic pathways in RNAseq

Category	Pathway	numDEInCat	numInCat	pvalue	Adj. pvalue
map00040	Pentose and glucuronate interconversions	24	33	9,50E-06	0,00083427
map00010	Glycolysis and Gluconeogenesis	31	47	1,77E-05	0,00083427
map04150	mTOR signaling pathway	167	355	1,90E-05	0,00083427
map04151	PI3K-Akt signaling pathway	165	353	3,31E-05	0,00109318
map00053	Ascorbate and aldarate metabolism	17	23	0,00015173	0,00400578
map00340	Histidine metabolism	18	26	0,00048152	0,01059352
map00270	Cysteine and methionine metabolism	19	29	0,00076854	0,01265965
map00500	Starch and sucrose metabolism	27	45	0,00082742	0,01265965
map00100	Steroid biosynthesis	11	14	0,00086316	0,01265965

a

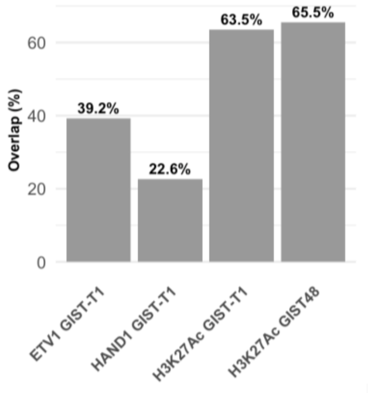

Factor	Genes overlapping with MITF	Total MITF Target Genes	Odds Ratio	P-value
ETV1 GIST-T1	813	2072	2.78	1.29e-96
HAND1 GIST-T1	469	2072	2.13	1.59e-38
H3K27Ac GIST-T1	1316	2072	3.20	1.60e-142
H3K27Ac GIST48	1191	1817	3.37	3.36e-136

b

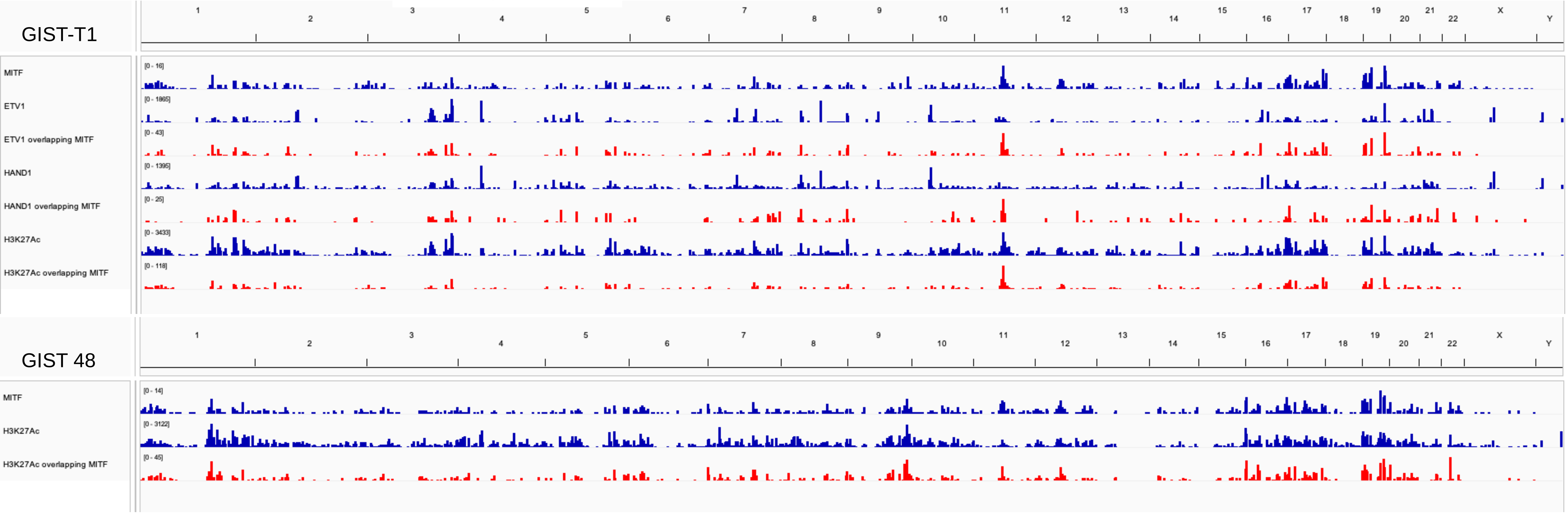

Supplementary Figure 1:MITF overlaps with ETV1, HAND1 and H3K27Ac. a) Barplot and table summarizing the overlap between MITF target genes and ChIP-seq peaks for ETV1, HAND1, and H3K27Ac in GIST-T1 and GIST48 cells. The barplot shows the percentage of MITF target genes that are also bound by each factor. The table includes the number of overlapping genes, total MITF targets, odds ratios (Fisher's exact test), and p-values. All comparisons show significant enrichment ($p < 0.001$). b) Genome-wide ChIP-seq peak profiles for MITF, ETV1, HAND1, and H3K27ac in GIST-T1 and GIST48 cells. Enrichment peaks were identified after peak calling, with Input used as the negative control. Blue peaks denote all regions significantly enriched in each dataset, while red peaks indicate sites overlapping MITF binding. Input was used for background normalization and is therefore not shown. The genome-wide overlaps reveal broad co-occupancy and suggest potential functional interactions between MITF and the other transcription factors or chromatin marks.

Supplementary figure 2: CHIPseq tracks of gene related to Lysosomes-autophagy.

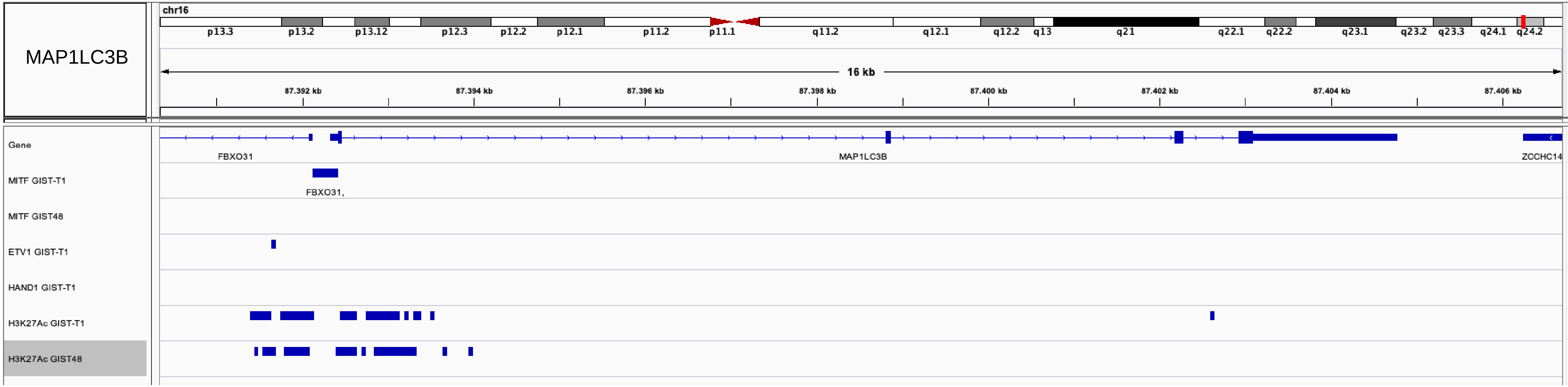

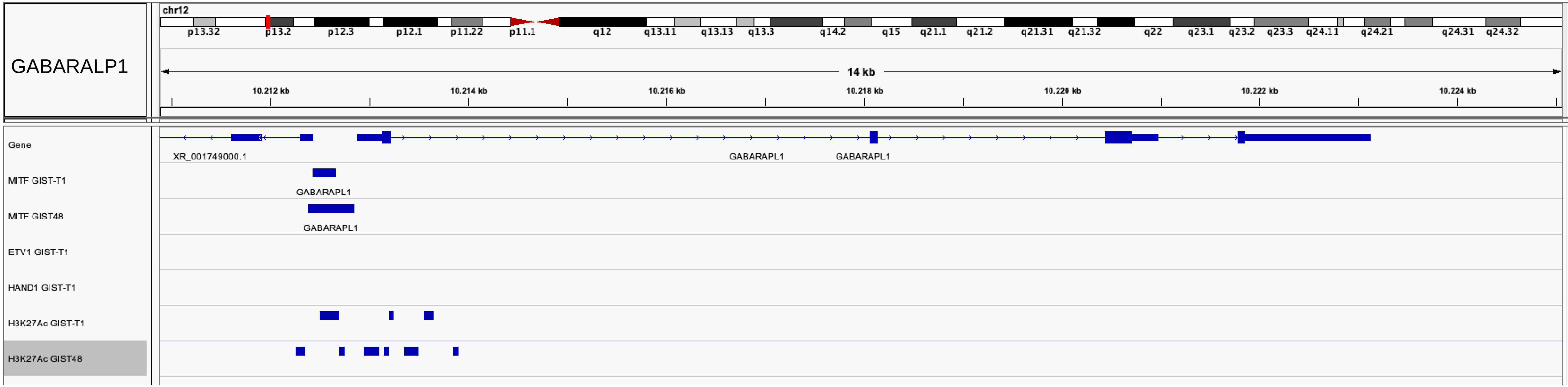

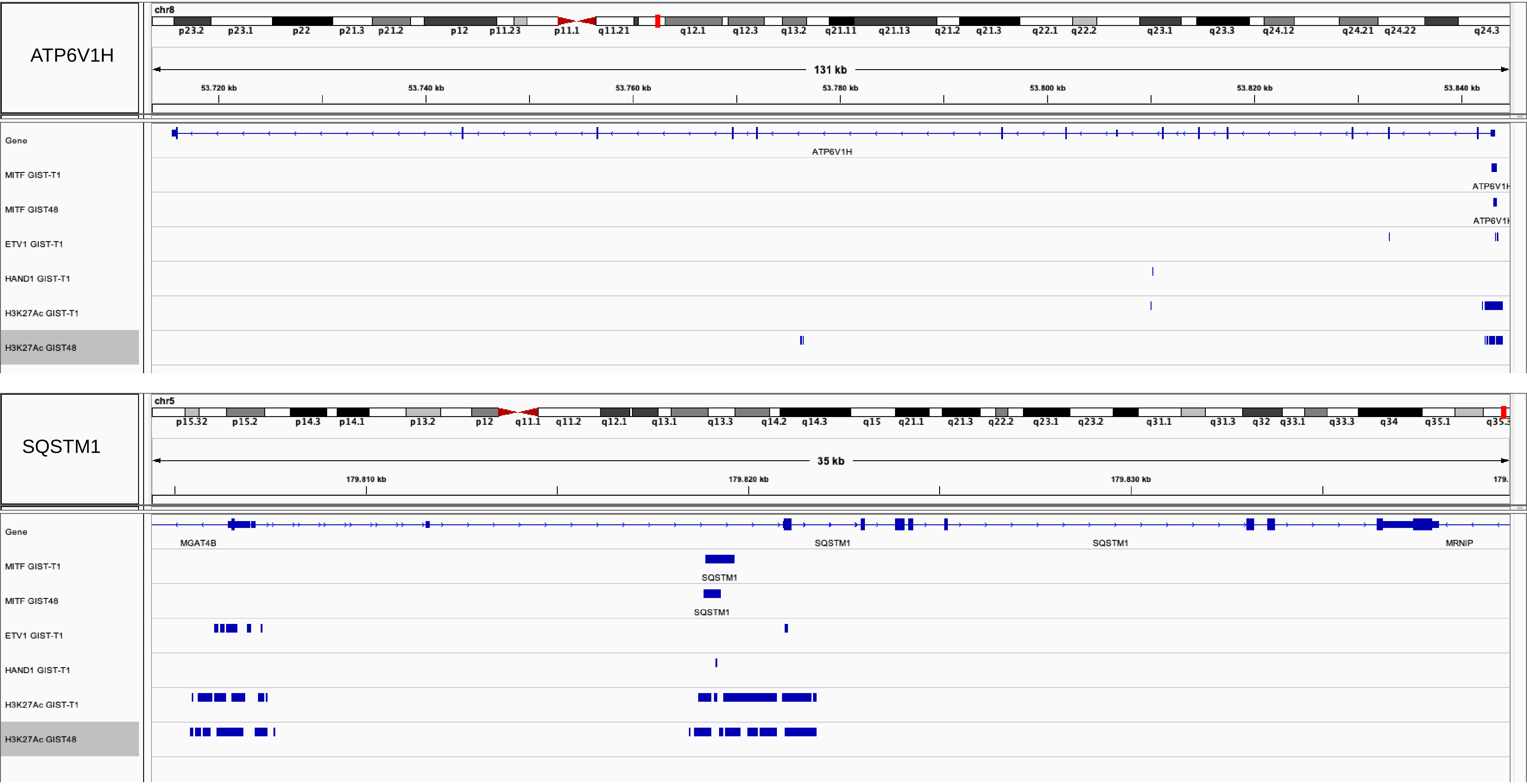
ATP6V1H
chr8
p23.2 p23.1 p22 p21.3 p21.2 p12 p11.23 p11.1 q11.21 q12.1 q12.3 q13.2 q21.11 q21.13 q21.2 q21.3 q22.1 q22.2 q23.1 q23.3 q24.12 q24.21 q24.22 q24.3
131 kb
53.720 kb
53.740 kb
53.760 kb
53.780 kb
53.800 kb
53.820 kb
53.840 kb
Gene
ATP6V1H
MITF GIST-T1
ATP6V1H
MITF GIST48
ATP6V1H
ETV1 GIST-T1
HAND1 GIST-T1
H3K27Ac GIST-T1
H3K27Ac GIST48
SQSTM1
chr5
p15.32 p15.2 p14.3 p14.1 p13.2 p12 q11.1 q11.2 q12.1 q13.1 q13.3 q14.2 q14.3 q15 q21.1 q21.3 q22.2 q23.1 q23.2 q31.1 q31.3 q32 q33.1 q33.3 q34 q35.1 q35.3
35 kb
179.810 kb
179.820 kb
179.830 kb
Gene
MGAT4B
SQSTM1
SQSTM1
MRNIP
MITF GIST-T1
SQSTM1
MITF GIST48
SQSTM1
ETV1 GIST-T1
HAND1 GIST-T1
H3K27Ac GIST-T1
H3K27Ac GIST48

Supplementary figure 2: CHIPseq tracks of gene related to Lysosomes-autophagy.

Genome browser tracks of MITF, ETV1, HAND1, and H3K27ac at autophagy-related gene loci in GIST-T1 and GIST48 cells. ChIP-seq enrichment peaks were identified after peak calling with Input as the negative control; Input was therefore used for background normalization and is not shown. MITF binds promoters or putative enhancers of MAP1LC3B, GABARAPL1, ATP6V1H, and SQSTM1, frequently overlapping with H3K27ac-marked active regulatory regions. In GIST-T1 cells, MAP1LC3B and GABARAPL1 display strong MITF peaks at their promoters, with MAP1LC3B additionally co-occupied by ETV1. ATP6V1H shows MITF binding at its promoter alongside distinct ETV1 and HAND1 sites, whereas SQSTM1 exhibits MITF peaks within an intragenic enhancer-like region, accompanied by ETV1 and HAND1 binding. These data indicate that MITF directly regulates key autophagy genes, with ETV1 and HAND1 contributing in a gene- and context-dependent manner.

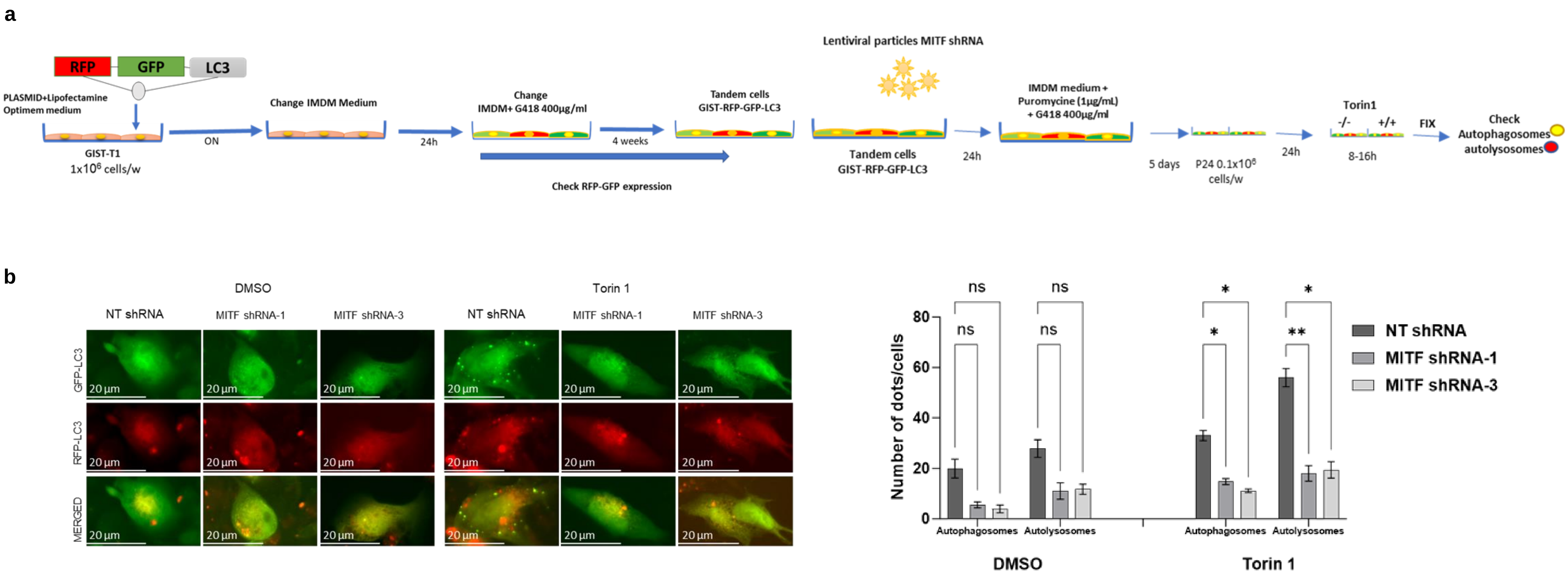

Supplementary Figure 3. Autophagosomes and autolysosomes are reduced in MITF-silenced GIST-T1 cells.
a) GIST-T1 cells stably expressing RFP-GFP-LC3 were transduced with non-targeting (NT) shRNA or MITF-targeting shRNAsh1 and shRNAsh3. After 24 hours, cells were treated with Torin 1 (0.2 µM) or vehicle control (DMSO). In this reporter system, both GFP and RFP signals are present in autophagosomes, whereas the acidic environment of autolysosomes quenches GFP fluorescence, leaving only the RFP signal. b) Representative immunofluorescence images of NT and MITF-silenced GIST-T1 cells treated with Torin 1 or DMSO. Quantification of autophagosomes (yellow puncta) and autolysosomes (red puncta) is shown. Data are presented as mean ± SEM. $*p < 0.05$, $**p < 0.01$ by one-way ANOVA followed by Bonferroni's post hoc test.

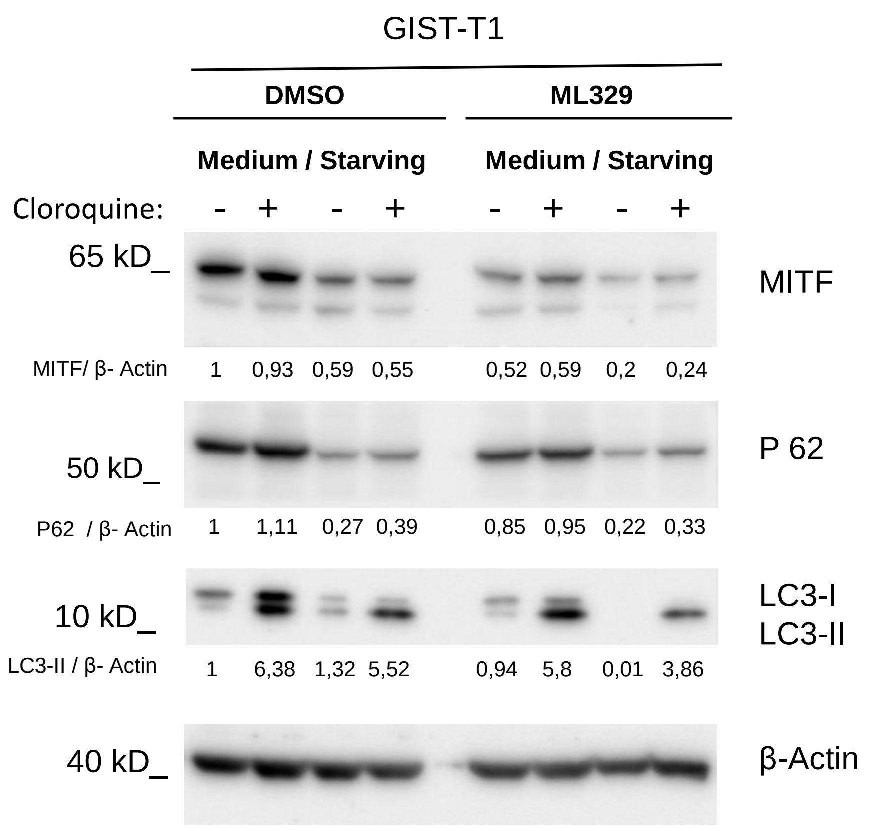

Supplementary Figure 4. **ML329 reduces LC3-II levels in GIST-T1 cells.**
GIST-T1 cells were treated with ML329 (5 μM) or DMSO (vehicle control) for 3 days. After treatment, cells were either maintained in complete medium or starved overnight in the presence or absence of chloroquine (10 μM) to block lysosomal degradation. Protein levels of MITF, LC3, and p62 were analyzed by Western blot using specific antibodies, with β-actin as a loading control. Densitometric quantification was performed as indicated. The image shows a representative blot from two independent experiments.

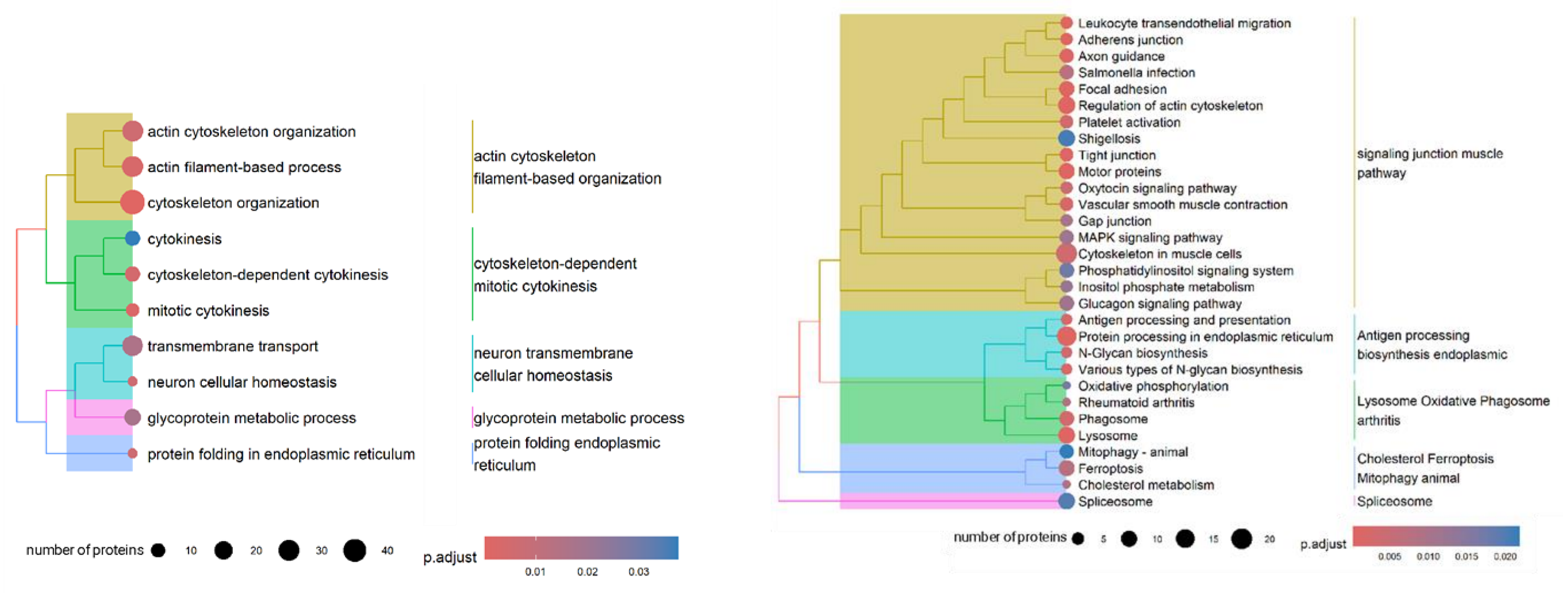

Supplementary Figure 5. Proteomic profiling of extracellular vesicles (EVs) from MITF-silenced GIST-T1 cells.
Gene Set Enrichment Analysis (GSEA) was performed on the proteomic dataset of EVs isolated from MITF-silenced cells. Enriched pathways were identified using Gene Ontology Biological Process (GO BP) terms (right panel) and KEGG pathway terms (left panel). Circle size indicates the number of proteins associated with each term, and numerical values represent enrichment scores or gene counts as specified.

FIGURA 3c

p-MITF:

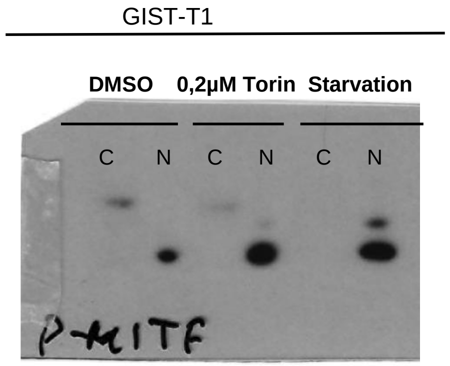

Lamin β1:

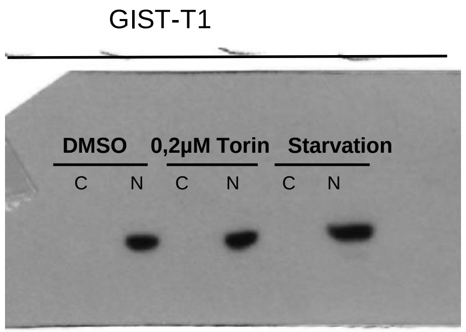

MITF:

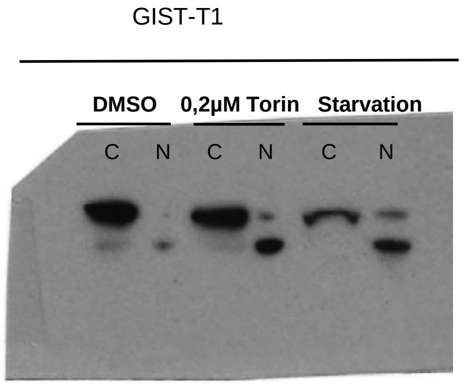

α -Tubulin:

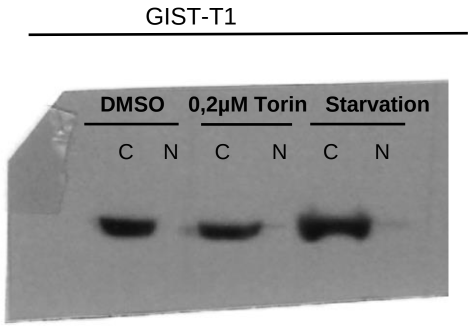

FIGURE 4 c

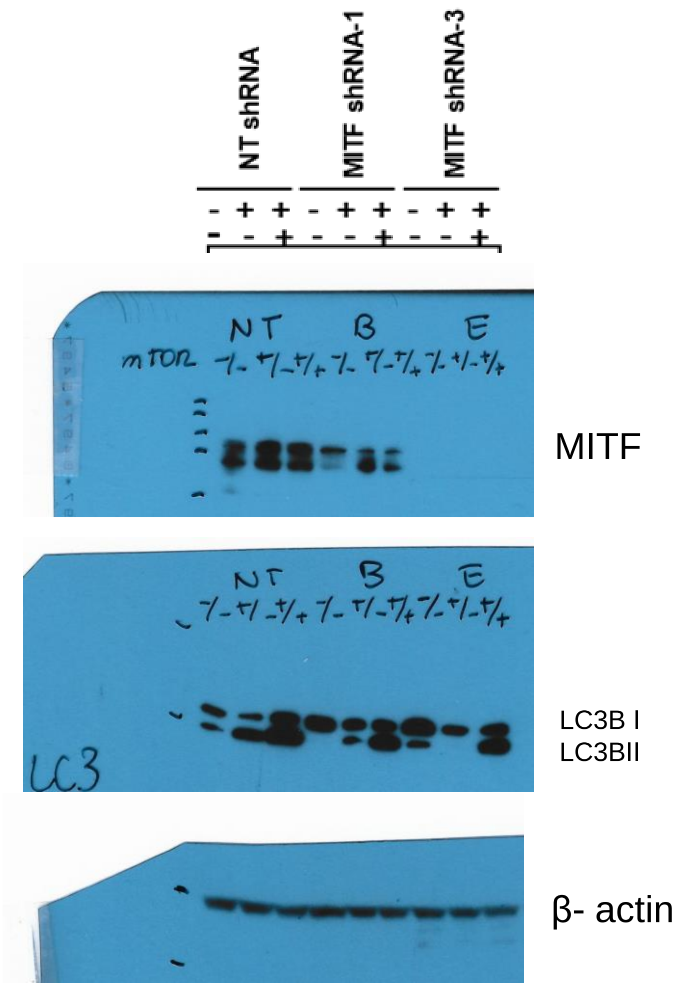

FIGURE 4 d

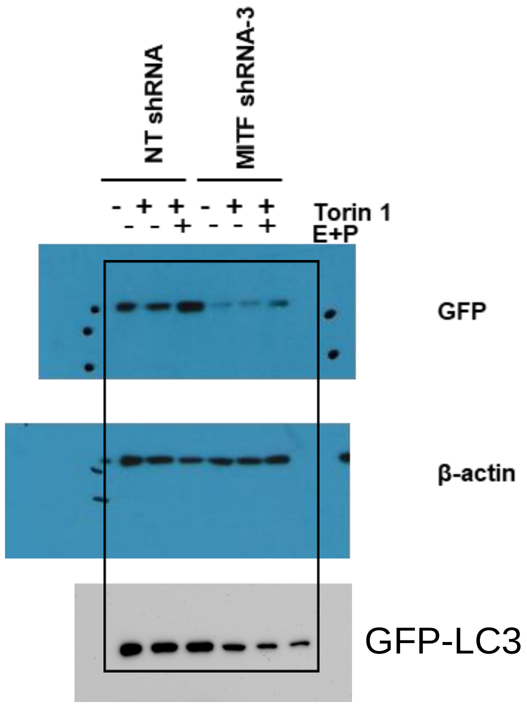

FIGURE 7

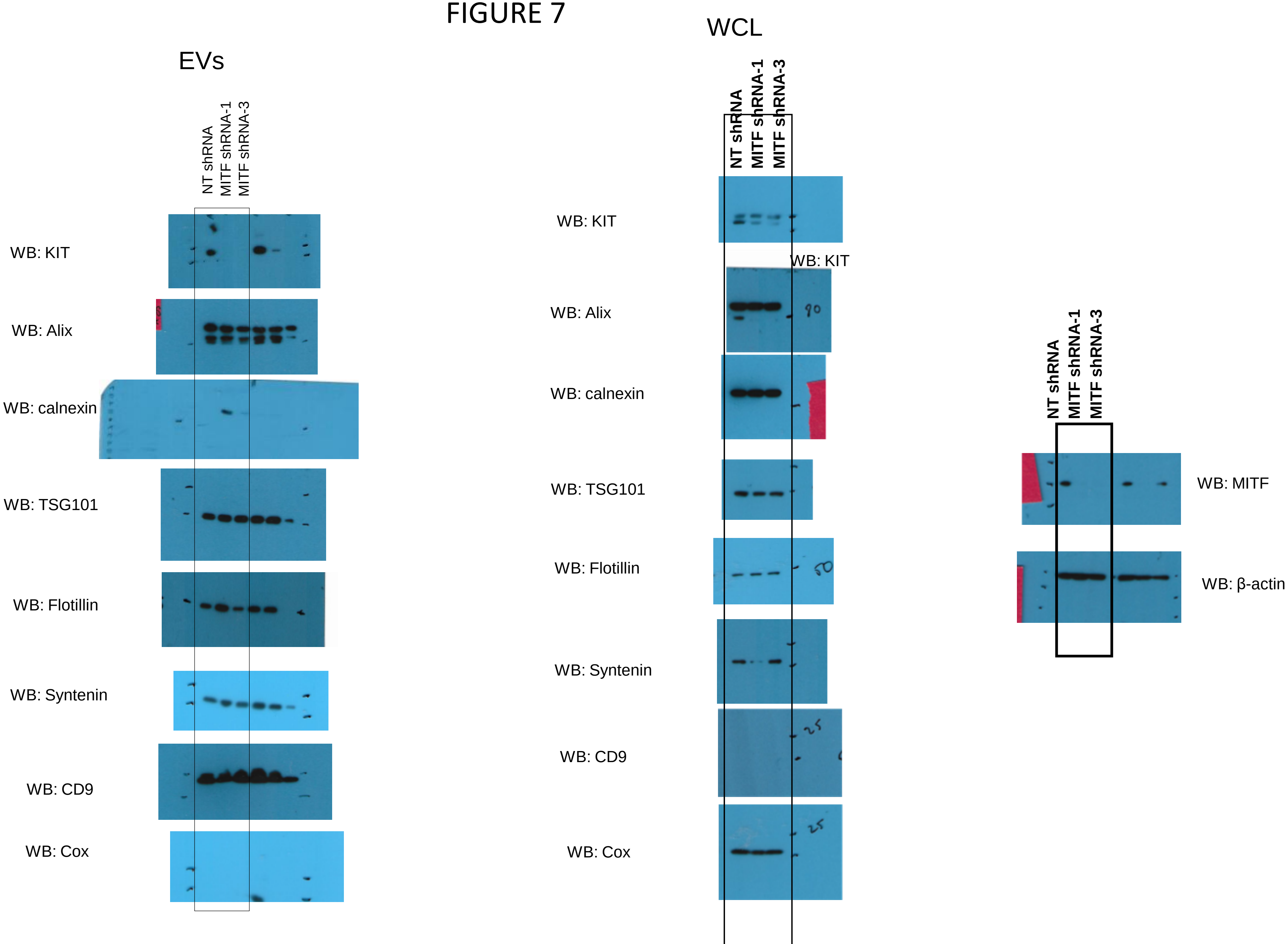
EVs
WCL
NT shRNA
MITF shRNA-1
MITF shRNA-3
WB: KIT
WB: Alix
WB: calnexin
WB: TSG101
WB: Flotillin
WB: Syntenin
WB: CD9
WB: Cox
WB: MITF
WB: β-actin

Supplementary Figure 4

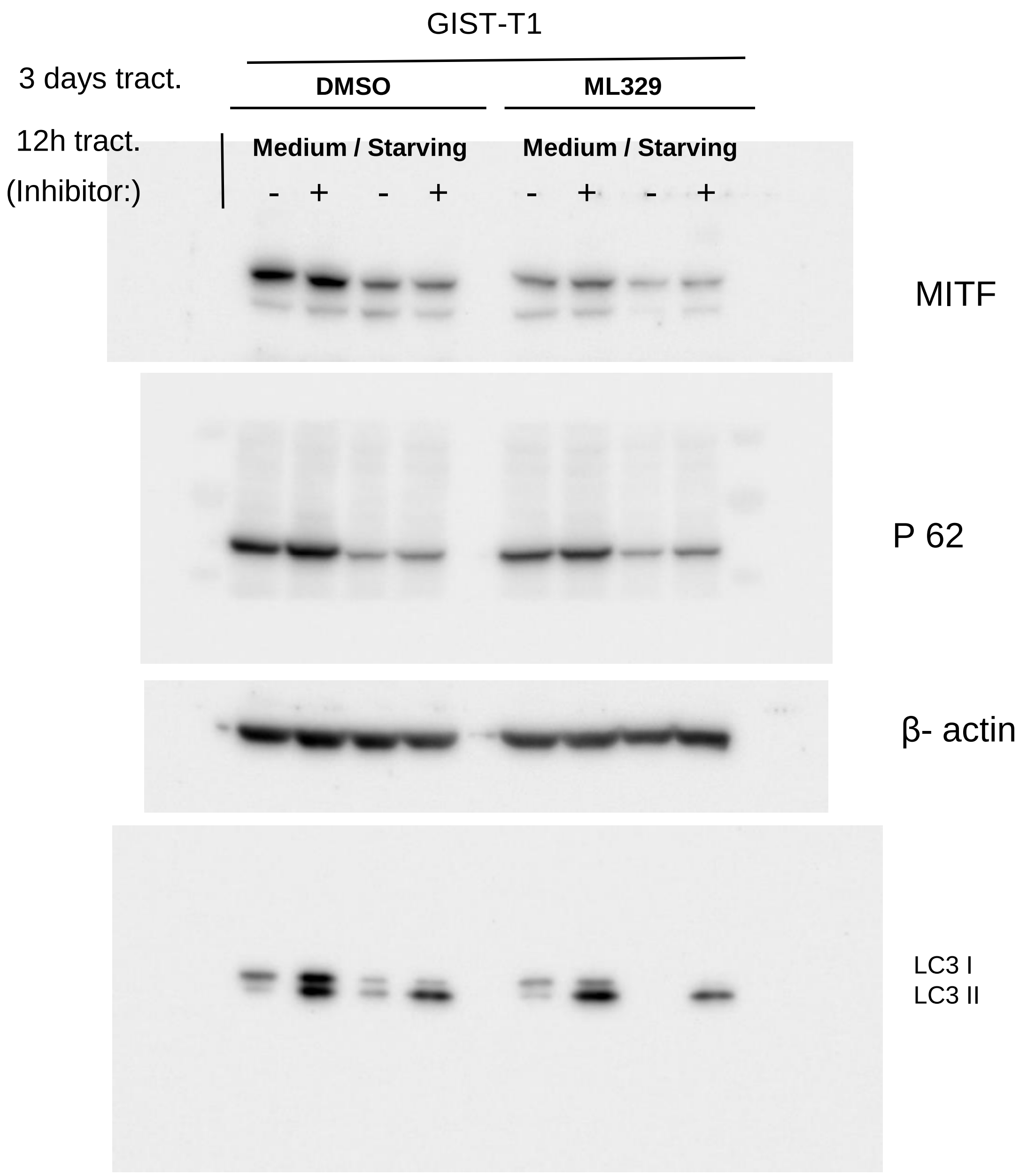

Mycoplasma test

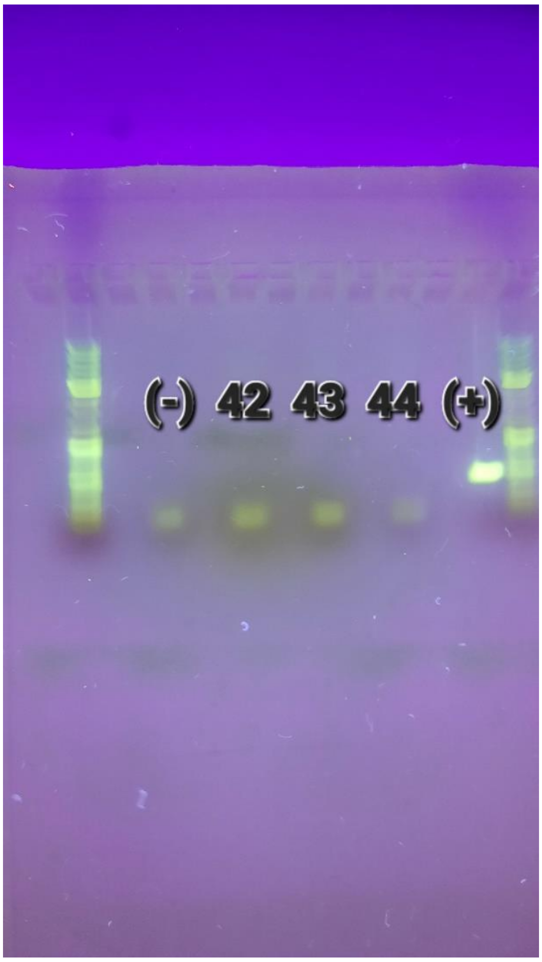

Lanes:

Ladder

(-): Negative control

42: GIST 882 Supernatants

43: GIST T-1 Supernatants

44: GIST 48 Supernatants

(+): Positive control

Ladder
